# Supplementary material for: Qualitative study of patient views on a ‘telephone-first’ approach in general practice in England: speaking to the GP by telephone before making face-to-face appointments
Source: BMJ Open. 2018 Dec 31;8(12):e026197. doi: 10.1136/bmjopen-2018-026197 (PMC6318515; doi:10.1136/bmjopen-2018-026197)
Supplement: Supplementary file 1 [file bmjopen-2018-026197supp001.pdf]

Appendix 1. Patient interview guide

This was a broad guide which was developed iteratively as early findings were reviewed by members of the research team

Introduction:

Please can you tell me a little bit about yourself, including whether you work

How long have you been at the practice?

How is your health in general? How regularly do you need access the GP?

Process of booking an appointment:

How do you make an appointment with your GP?

Has this changed since you have been a patient at the practice? (What used to happen?)

Have you had to call the surgery on behalf of someone else / or has someone else called on your behalf? How did that work?

When you call the surgery, how long does it take to get through? Have you experienced any difficulty in getting through?

Do you have to provide any information to the reception staff concerning the call? [could prompt here for how comfortable they feel speaking to receptionist?]

If offered a call back do you get it within a reasonable time period? Do you get to specify when you would like the call back? Have you had any difficulties around the call back?

Is it always a doctor that calls you back? If you have a preferred doctor, do you get to speak to them? [prompt on continuity of care]

Have there been any changes in how the telephone consultations have been run since they were introduced? Has that improved the service/ made it more difficult for you?

Your experience with telephone consultations:

How have you found the experience of using this system?

Is there anything about your lifestyle that makes it more or less difficult for you to use? [prompt: working hours, caring responsibilities etc]

What was the outcome of your last telephone consultation with the GP? (led to a face-to-face appointment, directed to another health-care professional such as nurse, or another service eg. Social services etc. or just given advice over the phone)

> *If not offered a face-to face appointment with GP:*
>
> Were you happy with this outcome? Do you feel the service was satisfactory? Did you seek care elsewhere instead? (e.g. at A&E)

If offered a face-to-face appointment:

If you have a preferred doctor, did you get to see him/her?

Have you noticed any change in the actual appointment [get more time with GP, GP better prepared etc]? Has the quality of the appointment changed?

Have you had other telephone consultations where something different happened? What was the outcome? [repeat prompts as above])

How you feel about the system and what it means for you:

What do you like about the current system for making appointments? ([prompt on convenience, chance to speak to GP when don't want appointment – reassurance, practice seems more organised, get an appointment when want one, appointments not being booked by people for unsuitable use]

What do you dislike about the current system for making appointments? [don't get an appointment when want one, can't communicate well using phone – lost personal connection, elderly – loss of social contact, safety concerns]

Is there anything you miss about the old system (e.g. being able to plan in advance)?

How do you feel about talking to the doctor on the phone? [prompts:Do you feel comfortable talking about your medical condition /do you have any concerns over confidentiality? Do you feel that you are able to make yourself understood?]

How did you feel about the system when it was first introduced? Has this changed over time? [i.e. have they got used to the system]

Has the telephone appointment system changed the way you seek health care services? [prompts: are they thinking about alternative services more before contacting GP? i.e. is this right for the GP should I be going to nurse or don't bother go straight to A&E?]

Has your contact with the GP surgery changed? (i.e. ring more often as know can speak to doctor etc). [prompt on whether offered an appointment, if not with GP who with? Just given advice over phone etc]

Concluding:

If you had the choice would you go back the old system or keep the new system? Why would you make that choice?

Do you think other patients share your view?

Overall are you happy with the care you receive at the practice?

Is there anything else about the telephone appointment system that you'd like to discuss that we haven't spoken about?

Thank you for taking the time to meet with me today
