# Supplementary material for: Qualitative study of patient views on a ‘telephone-first’ approach in general practice in England: speaking to the GP by telephone before making face-to-face appointments
Source: BMJ Open. 2018 Dec 31;8(12):e026197. doi: 10.1136/bmjopen-2018-026197 (PMC6318515; doi:10.1136/bmjopen-2018-026197)
Supplement: Supplementary file 2 [file bmjopen-2018-026197supp002.pdf]

Appendix 2: Characteristics of participants and the 'telephone first' approach

Specific characteristics of the 'telephone first' approach (from patient perspective)	ID	Age[1]	Gender[1]	Ethnicity[1]	Health status[1]	Employment status, nature of health concerns and frequency of GP use[1 2]	Approach preference[2]
**Practice 100 (urban, list size 5,000-9,999)** **Notable features:** no advance booking of face-to face appointments; patient can specify time for call-back; nurse practitioner triages some requests; choice of GP offered for call-back and face-to-face appointment; duty GP takes phone calls in reception office **Problems identified:** more difficult to see GP of choice on the day; can be difficult to get through to reception on Monday mornings **Previous system:** ring up to book in advance or queue up for same day appointments; same-day appointments often not available	100_1004	71	Female	white British	fair	Retired; multiple chronic health issues; frequent user of GP	'Telephone first'
	100_1006	79	Male	white British	good	Retired; multiple chronic health issues, infrequent user of GP	'Telephone first'
	100_1064	Adult[3]	Female	white British	fair	Carer for 85 year old mother with dementia; both have chronic health issues, frequent user of GP;	'Telephone first'
	100_1086	63	Male	white British	good	Recently retired; infrequent user of GP	'Telephone first'
**Practice 101 (urban, list size 5,000-9,999)** **Notable features:** possible to book telephone consultation in advance if preferred GP not available on the day; individual call back lists for each GP; prompt call-back or patient can specify time; some advance booking of face-to face appointments (for follow-ups or if patient unable to make same day appointment); nurse practitioner triages some requests **Problems identified:** can sometimes be difficult to get through to reception **Previous system:** ring up to book in advance; waited 2-3 days for appointment or longer for preferred GP	101_1002	76	Male	white British	very good	Retired; minor health issues requiring specialist input, infrequent user of GP; hearing impairment	'Telephone first'
	101_1006	65	Male	white British	very good	Full time carer for spouse; ongoing health issue requiring specialist input, infrequent user of GP	'Telephone first'
	101_1024	50	Female	other black	fair	Early retirement due to ill health; frequent user of GP	'Telephone first'
	101_1086	37	Male	white British	good	Works full time; ongoing mental and physical health issues; regular review by GP	'Telephone first'
**Practice 102 (urban, list size <5,000)** **Notable features:** quick response from reception to incoming calls; wait for call-back depends on urgency of the issue; some advance booking of follow-up appointments; nurse does some telephone consulting; some forward booking by GPs, patient can always see GP face-to-face if they wish – practice considering making further modifications. **Problems identified:** can sometimes be difficult to get through to reception but this is variable **Previous system:** ring up to book in advance; often waited 3-4 days for appointment but same day appointments available when required	102_1014	77	Female	white British	fair	Retired; multiple chronic conditions; frequent user of GP	'Telephone first'
	102_1019	67	Male	white British	poor	Retired; multiple chronic conditions; regular user of GP; seeing a specialist; lives alone	Conventional
	102_1031	47	Female	white British	poor	Works part time; ongoing mental and physical health issues; frequent user of GP; hearing impairment	'Telephone first'
	102_1064	65	Female	white British	good	Retired; infrequent user of GP	'Telephone first'

**Practice 103 (urban, list size 5,000-9,999)**<br>**Notable features:** receptionist asks patient whether issue is urgent – call backs prioritised dependent on urgency of issue; flexibility in scheduling call back – patient can request a call back on another day if preferred GP is not in; no advance booking of face-to-face appointments<br>**Problems identified:** can be difficult to get through to reception – phone line sometimes goes dead; face-to-face appointments not available if call later in the day requiring patient to call again the following day<br>**Previous system:** walk-in system for on the day appointments or book by phone – 2/3 days wait	103_1030	41	Female	white British	fair	Mother of two disabled children; frequent user of GP often for advice by phone	'Telephone first'
	103_1034	78	Male	white British	fair	Retired; very frequent user of GP	'Telephone first'
	103_1042	50	Female	white British	no response	Does not work; mental and chronic physical health problems; frequent user of GP	Conventional
	103_1053	71	Female	white British	good	Retired; frequent user of GP	'Telephone first'
	103_1074	67	Female	white British	fair	Retired; infrequent user of GP	'Telephone first', though with modifications
**Practice 104 (urban, list size <5,000)**<br>**Notable features:** receptionist asks patient for a reason for the call -GP reviews list of reasons given and offers face-to-face appointments to some patients on basis of this information alone (without speaking to patient directly); call-back within an hour by GP or by receptionist to call in for a face-to-face appointment<br>**Problems identified**: can be difficult to get through to reception on the phone on a Monday<br>**Previous system:** walk-in system	104_1070	54	Female	white British	fair	Does not work due to chronic health problems; infrequent user of GP as condition well controlled	'Telephone first'
	104_1087	74	Female	white British	good	Retired; increasing frequency of GP visits with age	'Telephone first'
**Practice 105 (urban, list size ≥ 10,000)**<br>**Notable features:** call-back within 30 minutes for urgent issues (wait for call-back depends on urgency); cut off time for patients to call by in order to receive same day call back (e.g. 16.30); nurse triage for some requests; choice of GP offered for call-back and face-to-face appointment; reception spread calls across all GPs, set number of calls per GP per day then a pooled list.<br>**Problems identified:** can be difficult to get through to reception on the phone; online booking no longer available<br>**Previous system:** booking in advance by phone – no difficulty getting an appointment but up to three week wait for non-emergency appointment; on line booking facility	105_1040	79	Female	white British	good	Retired; chronic health issues; frequent user of GP; hearing impairment	'Telephone first'
	105_1043	Adult[3]	Female	white British	n/a	Does not work – mother of young child; chronic health issues (self and child); frequent user of GP	'Telephone first'
	105_1090	78	Male	white British	fair	Retired; multiple chronic health issues; frequent user of GP	'Telephone first'
	105_1099	78	Male	white British	very good	Retired; fit and active; infrequent user of GP	'Telephone first'

**Practice 106 (urban, list size ≥ 10,000)**<br>**Notable features:** variable wait for call-back (from almost instant to many hours); choice of GP offered for call-back and face-to-face appointment; some flexibility for GP to book appointment for next day but no advance booking by reception (e.g. follow-up appointments); patients can choose time for call back.<br>**Problems identified:** can be difficult to get through to reception on the phone on a Monday; can wait all day for a call back<br>**Previous system:** booking in advance by phone – was beginning to get more difficult to get an appointment	106_1013	53	Female	white British	nr	Works flexibly from home; chronic health issue; anxiety; frequent user of GP; previous missed cancer diagnosis	Conventional
	106_1025	78	Female	white British	fair	Retired; chronic health issue; frequent user of GP	'Telephone first'
	106_1026	45	Female	white British	good	Not currently working due to ill health; infrequent user of GP	'Telephone first'
	106_1064	68	Female	white British	fair	Retired; chronic health issues but infrequent user of GP	'Telephone first'
	106_1077	61	Female	white British	fair	Does not work; mental health and multiple chronic physical health problems; frequent user of GP	'Telephone first'
**Practice 108 (urban, list size <5,000)**<br>**Notable features:** variable wait for call-back (from 30 minutes to many hours); duty GP takes calls all day, others only 8-11am; no advance bookings; recorded message indicates cut off time after which only emergency cases will receive a call back (e.g. 15.00)<br>**Problems identified:** variable reports regarding difficulty getting through on the phone; no longer offered choice of preferred GP; can wait all day for a call back; same-day call back not always available<br>**Previous system:** booking in advance by phone – was beginning to get more difficult to get an appointment	108_1032	59	Female	white British	good	Works full time but easy to take calls or make appointments; chronic condition; carer for elderly parents (with hearing impairment); frequent user of GP for self and as carer;	'Telephone first'
	108_1090	66	Female	white British	good	Retired; infrequent user of GP	Conventional
	108_1099	28	Female	Chinese	good	Student – some difficulty taking calls or making appointments; speaks English as a second language; unfamiliar with UK health system; frequent contact with GP	N/a (only experienced this system)
**Practice 110 (urban, list size 5,000 – 9,999)**<br>**Notable features:** phone lines shut off early in the day with recorded message to call the following day; no advance booking available; time of call-back not indicated; separate walk in system also reported to be in operation (bypassing phone system)<br>**Problems identified:** extreme difficulty getting through on the phone; if patient gets through appointments are often unavailable and patient is asked to call the following day; no longer offered choice of preferred GP; can wait all day for a call back;<br>**Previous system:** Advance booking system with long wait of a week or sit and wait on the day. Previously had online system but scrapped.	110_1007	60	Female	white British	fair	Early retirement due to ill health; frequent user of GP	Conventional
	110_1026	74	Male	white British	poor	Retired; multiple chronic conditions requiring specialist input; mental health issues; lives alone; reports limited user of GP due to Telephone	Conventional
	110_1095	63	Female	white/black	fair	Part time/voluntary work; ongoing mental health issues; reports limited user of GP due to Telephone	Conventional

**Practice 112 (urban, list size 5,000 – 9,999)**<br>**Notable features**: receptionist asks for brief details of issue – patient either put straight through to GP or receives very prompt call back; no advance booking available; separate system for nurse appointments<br>**Problems identified:** difficulty getting through on the phone – might take up to an hour; if patient calls after 9 am call backs are often unavailable and patient asked to call the following day; long wait in the surgery for booked appointment<br>**Previous system:** turn up at 8:00am and sit and wait on the day.	112_1015	65	Female	white British	good	Retired; infrequent user of the GP	'Telephone first'
	112_1046	Adult[3]	Male	other	n/a	Working parent,; speaks English as a second language	'Telephone first'
**Practice 114 (urban, list size 5,000 – 9,999)**<br>**Notable features:** receptionist does not ask about the nature of the issue (change from original system); receptionist provides indication of time for call-back and can schedule flexibly around patient's requirements; advance booking available for some follow-up appointments; nurse practitioner does some telephone consulting<br>**Problems identified**: system functioning well<br>**Previous system:** ring to book face-to-face appointment same day appointments were always available if required.	114_1008	48	Male	white British	good	Works/easy to take calls or make appointments; chronic health issues; frequent user of GP	'Telephone first'
	114_1029	Adult[3]	Female	white British	n/a	Carer for elderly father; works from home; frequent user of GP for self and as carer	'Telephone first'
	114_1058	72	Female	white British	poor	Retired; chronic health issues; frequent user of GP	Conventional
**Practice 117 (urban, list size 5,000 – 9,999)**<br>**Notable features:** prompt call-back from GP (often within 10-15 minutes – maximum 1 hour 30 minutes); no advance booking of face-to-face appointments; if preferred GP is not available patient offered choice to speak to a different GP or ring back when available; call back only available for emergencies after 16.00<br>**Problems identified:** time cut off to ensure face-to-face appointment available on the day is unclear;<br>**Previous system:** same day appointment system - rang on the day and had to see whoever was available that day or ring the next day. Sometimes a long wait to see Dr of choice	117_1027	51	Female	white British	very good	Works/difficult to take calls; infrequent user of GP;	'Telephone first'
	117_1029	60	Female	white British	poor	Does not work due to ill health and caring responsibilities; multiple chronic conditions; very frequent user of GP	'Telephone first'
	117_1066	32	Female	white British	good	Single mother/part time voluntary work; infrequent user of GP	'Telephone first'
	117_1073	86	Male	white British	good	Retired; recent hospital stay but previously in good health; infrequent user of GP	'Telephone first'

[1] Based on patient survey
[2] Based on interview data
[3] Interviewee completed survey on behalf of another patient (age of interviewee not given)
