# Supplementary material for: Qualitative study of patient views on a ‘telephone-first’ approach in general practice in England: speaking to the GP by telephone before making face-to-face appointments
Source: BMJ Open. 2018 Dec 31;8(12):e026197. doi: 10.1136/bmjopen-2018-026197 (PMC6318515; doi:10.1136/bmjopen-2018-026197)
Supplement: Author's manuscript [file bmjopen-2018-026197.draft_revisions.pdf]

BMJ Open is committed to open peer review. As part of this commitment we make the peer review history of every article we publish publicly available.

When an article is published we post the peer reviewers' comments and the authors' responses online. We also post the versions of the paper that were used during peer review. These are the versions that the peer review comments apply to.

The versions of the paper that follow are the versions that were submitted during the peer review process. They are not the versions of record or the final published versions. They should not be cited or distributed as the published version of this manuscript.

BMJ Open is an open access journal and the full, final, typeset and author-corrected version of record of the manuscript is available on our site with no access controls, subscription charges or pay-per-view fees (http://bmjopen.bmj.com).

If you have any questions on BMJ Open's open peer review process please email info.bmjopen@bmj.com

BMJ Open

A qualitative study of patient views on a 'telephone-first' approach in general practice: speaking to the GP by telephone before making face-to-face appointments

Journal:	*BMJ Open*
Manuscript ID	bmjopen-2018-026197
Article Type:	Research
Date Submitted by the Author:	21-Aug-2018
Complete List of Authors:	Ball, Sarah L.; RAND Europe, Cambridge Centre for Health Services Research<br>Newbould, Jennifer ; RAND Europe<br>Corbett, Jennie; RAND Europe<br>Exley, Josephine; London School of Hygiene and Tropical Medicine<br>Pitchforth, Emma; RAND Europe,<br>Roland, Martin; University of Cambridge, GP and Primary Care Research Unit
Keywords:	General practice, Remote consultation, Appointments and schedules, Telephone, Patient satisfaction

SCHOLARONE™
Manuscripts

For peer review only

A qualitative study of patient views on a 'telephone-first' approach in general practice: speaking to the GP by telephone before making face-to-face appointments

Sarah Ball [1], Jennifer Newbould[1], Jennie Corbett[1], Josephine Exley[1], Emma Pitchforth[1 3], Martin Roland[2].

[1] Cambridge Centre for Health Services Research, RAND Europe, Westbrook Centre, Cambridge CB4 1YG, UK

[2] Cambridge Centre for Health Services Research, Institute of Public Health, University of Cambridge, Forvie Site, Cambridge CB2 0SR, UK

[3] University of Exeter University of Exeter Medical School, St Luke's Campus Heavitree Road, Exeter EX1 2LU, UK

Correspondence to: Sarah Ball, Cambridge Centre for Health Services Research, RAND Europe, Westbrook Centre, Cambridge CB4 1YG, UK, Email - sarahb@rand.org, Telephone - +44 1223 353 329 x 2594

Author email addresses:

Jennifer Newbould - newbould@rand.org

Jennie Corbett - jenniecorb@gmail.com

Josephine Exley - Josephine.Exley@lshtm.ac.uk

Emma Pitchforth - E.Pitchforth@exeter.ac.uk

Martin Roland - mr108@medschl.cam.ac.uk

Abstract

Objective: To understand patients' views on a 'telephone-first' approach, in which all appointment requests in general practice are followed by a telephone call from the GP, and to identify the characteristics of practices and patients that appear to influence the acceptability of the approach

Design: Qualitative interviews with patients and carers

Setting: Twelve general practices in England

Participants: 43 patients, including 30 women, nine aged over 75, four parents of young children, five carers, five patients with hearing impairment and two whose first language was not English.

For peer review only - http://bmjopen.bmj.com/site/about/guidelines.xhtml

For peer review only

Results: Patients expressed varied views, often strongly held, ranging from enthusiasm for to hostility towards, the 'telephone-first' approach. The new system suited some patients, avoiding the need to come into the surgery, but was problematic for others e.g. when it was difficult for someone working in an open plan office to take a call-back. A substantial proportion of negative comments were about the operation of the scheme itself rather than the principles behind it, for example difficulty getting through on the phone or being unable to schedule when the GP would phone back. Some practices were able to operate the scheme in a way that met their patients' needs better than others and practices varied significantly in how they had implemented the approach.

Conclusions: The 'telephone-first' approach appears to work well for some patients but others find it much less acceptable. Some of the reported problems related to how the approach had been implemented rather than the 'telephone-first' approach in principle and suggests there may be potential for some of the challenges experienced by patients to be overcome.

265 words

Key words

General practice

Remote consultation

Appointments and schedules

Telephone

Patient satisfaction

Strengths and limitations of this study

Participants included a wide range of patients and carers from a diverse group of practices.

- Patients and carers selected for interview had recent experience of the telephone-first approach.
- Participants were purposively sampled to include a wide range of views on the new approach.
- Semi-structured interviews allowed participants to discuss in detail their own experiences of the telephone first approach
- Practices agreeing to take part in the study may have been operating the telephone-first approach more successfully than those that declined.

Introduction

Increasing demand for general practice care is leaving practices in the UK struggling to meet patient need.[1] In response, some practices (at least 150 in England) have adopted a novel 'telephone-first' approach to managing patient requests for a consultation. In this whole system approach, all appointment requests are followed by a telephone call from the general practitioner (GP). Either the

For peer review only - http://bmjopen.bmj.com/site/about/guidelines.xhtml

For peer review only

issue is resolved during this call through provision of advice, a prescription or redirection to another health professional, or the patient is invited for a face-to-face consultation, usually on the same day.

Currently, two commercial companies (Dr First and GP Access) promote this approach in the UK and provide management support to practices adopting it. The approach has been advocated by NHS England based on significant benefits reported by the companies including improved access to primary care, improved patient satisfaction and reductions in both primary and secondary care utilisation.[2] However, an independent evaluation that we carried out found no evidence of an overall reduction in GP workload, no evidence of reduced secondary care costs and, while patients were able to be seen much more quickly, there was little overall improvement in patient satisfaction as expressed in patient surveys.[3]

While published studies on patient satisfaction with GP telephone consultations in general report positive findings[4,5,6], the telephone-first approach is a much more fundamental innovation in service provision and the National Association for Patient Participation has raised a range of concerns and opposition to the approach.[7] The patient surveys described in a previous paper [3] elicited a wide range of views about the telephone-first approach, from strongly positive to strongly negative. In this paper, we report the findings of qualitative interviews conducted with patients and carers to explore these views in greater depth.

Methods

Site selection, sampling and recruitment

Qualitative interviews with patients were undertaken in twelve GP practices using the telephone-first approach. Participating practices came from areas of England including the North East, North West, Midlands, East Anglia, London, the South East and the South West. Practices were selected purposively from the twenty practices participating in a patient and carer survey as part of our wider evaluation,[2] to include those with a range of experiences of adopting the 'telephone-first' approach, including practices reporting positive experiences and those that had experienced or overcome problems.

In the first instance, patients who were potential participants indicated their interest in being contacted for an interview by returning a reply slip that accompanied the patient and carer survey. Purposive sampling of those who expressed an interest was carried out by the research team, to gain a range of views and to ensure people with the following characteristics were included: older people, people who work, people with disabilities, people with chronic conditions and those with English as a second language. Selected interested participants were contacted by a member of the research team by the preferred contact mode indicated in the reply slip (telephone or email) and invited to take part in an interview.

Data collection

Semi-structured interviews were conducted by four researchers (SB, JN, JC, JE), either at the patient's home or at their GP surgery, as requested by the patient. All interviewees gave written consent to be interviewed. A common interview guide, informed by the literature, was used for each interview (see appendix), although emphasis was given to allowing participants to talk from their

For peer review only - http://bmjopen.bmj.com/site/about/guidelines.xhtml

own perspective and elements of the guide were developed iteratively as the study progressed. The main focus of the interview was on patients' and carers' views of the advantages and disadvantages of the 'telephone-first' approach including its convenience, perceptions of quality of care and impacts on the doctor-patient relationship. Interviews were audio-recorded with the participants' permission, and transcribed verbatim. Transcripts were anonymized by removing references to identifiable names and places.

Data analysis and reporting

Data analysis proceeded in parallel with data collection and informed the iterative development of the interview topic guide and observation protocol. Thematic analysis of the data was conducted based on the principles outlined by Boyatzis.[8] Transcripts were read and re-read and 'codes' applied to meaningful sections of text. Coding was conducted by SB, JC, JE, JN and EP. As analysis progressed codes were grouped into overarching or organising themes using NVivo 10 software. Data within themes were scrutinized for confirming and disconfirming views across the range of participants. Emerging findings were shared and discussed regularly within the study team. In addition to understanding patients' views and experiences of the 'telephone-first' approach we sought to identify characteristics of both practices and patients which appeared to have influenced its acceptability to patients. We have followed SRQR reporting guidelines.[9]

Patient involvement

A study steering group was established, which included four patients along with healthcare professionals. The steering group met on three occasions and provided input into the design and conduct of the study including advice on patient materials produced during the study. Patient representatives from the steering group and those from participating practices attended a learning event at which practices shared their experiences of the 'telephone-first' approach and commented on our findings to inform their interpretation.

Results

Interviews were conducted with 43 patients and carers registered at 12 GP practices across England, all of which had been using the 'telephone-first' approach for between 18 months and 5 years. Respondents were aged between 28 and 86 years and included older people, parents of young children, carers, working people and a number of other 'hard to reach' groups (Table 1). The practices at which the patients were registered varied with respect to: list size, geographical location and a range of characteristics of the catchment population, such as deprivation and ethnicity. The characteristics of these practices and further details on the characteristics of the patients and carers interviewed are outlined in Appendix 1.

Characteristics	Number of interview subjects (%)

For peer review only - http://bmjopen.bmj.com/site/about/guidelines.xhtml

Female	30 (69.8%)
Aged over 75	9 (20.9%)
Parent of child under 13 years	4 (9.3%)
Carer[1]	5 (11.6%)
Working	11 (25.6%)
Hearing impaired	5 (11.6%)
First language not English	2 (4.7%)
Living with a chronic condition	24 (55.8%)
**Total**	**43**

[1] All five carers were interviewed in both their capacity as a carer and as a patient

Table 1. Characteristics of interview participants

Interviews provided a rich source of data, and patients were open in expressing their views (ranging from enthusiasm to hostility towards the approach), describing their experiences and reporting on a range of perceived advantages and disadvantages. The findings are structured around two main questions. First, which aspects of the approach were acceptable or unacceptable to patients, and secondly which factors influenced the acceptability of the approach.

Overall acceptability of the 'telephone-first' approach

While the majority of patients, when asked to make a choice, said that they would stick with the 'telephone-first' approach rather than return to the system that their practice previously ran, their responses were nonetheless extremely varied: some patients reported being highly satisfied, others found the approach unacceptable.

Several gave strong endorsements, including one who commented that:

> *Apart from just jumping in the car and going walking into a doctor's, there's no other way you could improve that.* (101_1002 – Male patient in his 70s, retired, minor health issues requiring specialist input, hearing impairment)

In contrast, a small number of patients reported being so dissatisfied with the approach that they were considering moving to a different practice:

> *I just don't like it [the 'telephone-first' approach]. […] I just want a doctor's where I can go in, phone up, whatever which way I want to do it, book an appointment and go. […] I don't feel like I've got any help anywhere.* (103_1042 – Female patient in her 50s, not in employment, mental and chronic physical health problems)

Others were ambivalent, considering the new approach to be the least bad option:

> *I'm prepared to stick with it because, I mean, going back to the old system, no. That's even worse.* (106_1077 – Female patient in her 60s, not in employment, mental health and multiple chronic physical health problems)

For peer review only - http://bmjopen.bmj.com/site/about/guidelines.xhtml

In describing their experiences of the approach, patients outlined a broad range of advantages and disadvantages in relation its impact on how they were able to access care and the nature or quality of the care received. These fell into a number of distinct categories considered in turn below.

Acceptability of the booking process

While several patients described how the initial contact with the practice to request an appointment was more streamlined following the changes, a greater number reported difficulties with or objections to the new booking process (such as long waits for calls to be answered, restricted opening times for telephone lines or a lack of clarity around how the system worked). One patient, among a number who reacted with hostility to the introduction of the approach, described a situation in which it had taken days to get to speak to a GP:

> *…. tried for two days, press five [for automatic redial] still off - and on the Thursday someone actually answered. […] Said 'what is it?' so I said what [was wrong] and I need to see the Doctor. They phoned me back then. She says well Doctor [name redacted] is not in today - phone tomorrow. Bump [phone being hung up]. So I phoned the next morning 8 o'clock. Phones off. I phoned every five minutes till 8.30am - it came on, 'surgery's now full', phone Monday. […]You should try the system… It's that bad you couldn't make it up. If they had someone to report it to I'd prosecute them. They're terrible.* (110_1026 – Male patient in his 70s, retired, multiple chronic conditions and mental health issues)

Several patients reported having a lack of awareness of how the approach would work in practice at the outset, and were unhappy with a lack of consultation around its introduction, which led to confusion, anxiety and misconceptions regarding the purpose behind the introduction of the approach. A similar number of patients however, commented that their initial misgivings had not, by and large, been realized. The degree to which patients reported that they had been consulted (or at least informed) ahead of the introduction of the new approach also varied considerably.

Responsiveness of the approach

More than half the patients interviewed commented on the prompt response of GPs following their initial call to the surgery. Guaranteed same-day call-backs (in some cases within minutes or within an agreed time slot) reassured patients who were anxious about what might be wrong with them, and the availability of timely face-to-face appointments (if required) was appreciated by many:

> *This way I find if he [the GP] deems it serious enough for you to call in to see him, he'll see you the same day, which is brilliant.* (100_1004 – Female patient in her early 70s, retired, multiple chronic health issues)

Patients at some practices however, described a delayed or unpredictable response, with no indication as to when the doctor would call back or a lack of availability of appointments after speaking to the doctor. Variability between the reports of patients at different practices suggests differences in the way the call-back system was managed. Several patients indicated that they were happy to wait for a call back, in the knowledge that calls were prioritized according to need:

For peer review only - http://bmjopen.bmj.com/site/about/guidelines.xhtml

> *I mean sometimes if he's [the GP] really busy, you don't hear from him for a couple of hours but then he's obviously got patients there that are a priority. They know how to prioritize them which is good.* (102_1014 – Female patient, in her late 70s, retired, multiple chronic conditions)

Several other patients, however, described finding this unpredictability particularly difficult, including a patient whose job as a support worker meant that she was unable access her mobile phone during a shift and two patients with mental health issues who reported feeling distressed while they waited for a response from the GP. A few patients, whilst commenting on the inconvenience of having to wait, acknowledged that this had not been a particular issue for them, but indicated concern that it would be an issue in case of an urgent need.

Equitable / fair access to care

More than a quarter of patients interviewed indicated that they appreciated that the 'telephone-first' approach led to more efficient use of resources and improved access for patients with the greatest need for urgent care, and several recognized that this in turn conferred benefits to them as individuals (ensuring prompt access if required):

> *you get to speak to a doctor before you go in for your appointment, because I think there are a lot of times when you actually don't need to see a doctor face to face but, sometimes the advice of a doctor can put your mind at ease or just give you the information that you need to know - so then, you are not wasting your time and you are not wasting their time.* (117_1066 – Female patient in her 30s, single mother in part time work, infrequent user of GP)

> *It's better this way because then you don't get any timewasters. […] Then you haven't got to wait. They put you first before the timewasters.* (105_1043 - Female patient, mother of young child, both with chronic health issues)

Patients differed in their perceptions of the intended function of the approach with respect to redirecting patient demand. While some patients perceived the 'telephone-first' approach to be a fair system for meeting patient need, others saw it as a barrier, intended to keep as many patients as possible away from face-to-face appointments with busy doctors:

> *It certainly feels like a gate-keeping service […] like being kept as much at arm's length as possible.* (114_1058 –Female patient in her early 70s, retired, chronic health issues)

Several patients described feeling a requirement to justify their requests to see a doctor face-to-face, needing to *'fight and […] protest to have an appointment to see a doctor'* (103_1042 - Female in her 50s, not in employment, mental and chronic physical health problems). Several others expressed concern on behalf of vulnerable patients, such as the elderly or those with mental health issues, who may lack the confidence or communication skills to push for an appointment when required.

Convenience and flexibility of the approach

For peer review only - http://bmjopen.bmj.com/site/about/guidelines.xhtml

For peer review only

More than half the patients interviewed reported that they found that the approach enabled more convenient access to advice and care than the system previously in place, with benefits including: being able to get on with daily activities rather than having to wait for long periods in the surgery (facilitated by the availability of mobile telephone contact); reduced need to travel to the surgery unnecessarily (a particular benefit for those whom travelling to the surgery was difficult, such as a mother with disabled children, a carer whose husband was disabled with chronic conditions and mobility issues, and those dependent on limited public transport services); and access to medication without the need for a face-to-face appointment:

> *I like the fact that on a day like today, it is chucking it down, it's miserable, it's cold, if my mum had had to come to the doctor instead of a phone call on any day where the weather was like this, it would have caused her a lot of pain.* (102_1031 – Female patient in her 40s, works part time, ongoing mental and physical health issues)

The remainder, however, found the approach inconvenient in one or more respects, including: not being able to book appointments in advance; receiving a call from the GP at inconvenient times (when shopping, on public transport, or at work); or having to stay at home to wait for a call, particularly if it related to a personal issue that it was difficult to discuss in public:

> *you can't sit glued to your phone all day waiting for a call, even if you've got a mobile phone, you might be in the shower, or you might be in a shop or on the other phone or something. So it doesn't work… and how people who are working, expect to get an appointment I don't know really.* (110_1007 – Female patient, early retirement due to ill health)

Patients at several practices, described how such issues had been addressed by ensuring flexibility in the approach, such as by accommodating patient requests for a call-back at a particular time or offering limited advanced bookings for those unable to attend on the same day.

Similarly, a number of patients with particular difficulties that had an impact on how they were able to interact with the practice using the 'telephone-first' approach, described how such difficulties were overcome by minor adjustments and a flexible approach e.g. special arrangements for patients whose first language was not English, or those with a hearing impairment.

Communication by telephone and the nature of the telephone consultation

Patients described advantages and disadvantages of initially consulting by telephone rather than face-to-face. While some patients described feeling very comfortable communicating by telephone, including two patients with mental health issues who preferred telephone consultations because they felt more relaxed, others reported difficulties describing symptoms or understanding and recalling the GP's advice. Several patients reported that they felt anxious when communicating on the telephone (including older people, those with mental health issues, hearing impairment and one for whom English was not their first language), or reported concerns on behalf of other patients:

> *I've got a friend, an old lady who's 88, going on 89, I think, and she absolutely hates [it]. She says "I can't talk on the phone, I just don't know what to say, I just go to pieces." And somebody like her, it's just totally awful you know, it's not satisfactory at all.* (110_1007 – Female patient in her 60s, early retirement due to ill health)

For peer review only - http://bmjopen.bmj.com/site/about/guidelines.xhtml

A number of patients (the majority of whom were in there 50s or 60s with chronic conditions or mental health issues), commented that they found the approach to be impersonal – rushed and to the point. This was in part attributed to a lack of relational continuity of care (see below) but also due to the nature of the telephone consultation itself, and the absence of the social cues present in face-to-face interaction. One patient with mental health issues described the negative impact of a lack of face-to-face contact on the nature of the consultation:

> *I just cannot cope with not seeing someone's face […] I just want to speak in a room with the door closed face-to-face with someone so that I can be honest about how I am feeling and what's been happening lately. So I don't really say much over the phone […] whereas if it was face-to-face I would explain more* (110_1095 – Female patient in her 60s, part-time work, ongoing mental health issues)

Changes to the nature of face-to-face appointments

While the majority of patients did not report changes in the nature of face-to-face appointments following the introduction of the 'telephone-first' approach, a small number noted improvements such as reduced waiting time in surgery and a calmer more relaxed atmosphere, with patients experiencing less time-pressure during appointments. Some patients suggested that the approach led to GPs being better prepared and the appointment being more streamlined as a result. A few among those who did not observe any difference in the nature of face-to-face appointments, however, commented that having to repeat details given over the telephone in the face-to-face appointment was an annoyance and appeared inefficient.

Continuity of care

Given claims made by commercial providers that the 'telephone-first' can improve continuity of care for patients, interviewees were asked specifically about changes in the ease with which they were able to see a preferred GP. A small number of patients reported finding it easier to see or speak to their preferred GP than with the previous system, as a result of the way in which calls were allocated within the practice, with patients being able to specify which GP they would like to call them back. If this was not possible, they could request a face-to-face appointment with the preferred GP during the telephone call. A significant number, however, reported the opposite and found it harder to see their GP of choice, observing a trade-off between being seen or spoken to quickly and seeing their preferred GP.

Several patients expressed concern about whether an unfamiliar GP could effectively assess an issue over the telephone and worried about the lack of opportunity to develop or sustain a relationship with a GP (a particular concern among patients with chronic conditions and those with ongoing mental health issues):

> *[an unknown] GP rang me back and I wasn't sure whether he knew anything about me. I'm quite sure he'd looked at my records very briefly but I was concerned because it's quite complicated and my preferred GP knows from day one and has worked with me and referred me and supported me, so I didn't know how much this person knew and I just was a little bit unsure and a little bit anxious about whether or not he knew enough about me* (110_1095 – Female in her 60s, part-time work, ongoing mental health issues)

For peer review only - http://bmjopen.bmj.com/site/about/guidelines.xhtml

Patient safety

Patient views on the impact (or potential impact) of the 'telephone-first' approach on patient safety also varied considerably between patients and across practices (with around a third expressing some concerns). While some patients felt vulnerable because of difficulties getting through to the practice by telephone or that diagnoses might be missed in telephone consultations, others thought the approach was safer for patients, in part because of the considerable reduction in waiting times for appointments:

> *Well I think you get to talk to your doctor when you need to talk to him or her, rather than having a long wait and perhaps getting progressively worse. Certainly if it's an acute condition, it can make a difference, can't it?* (100_1004 - Female patient in her early seventies, retired, multiple chronic health issues)

> *So I phoned up and it was early in the morning and I mentioned to the receptionist what the problem was, and so within minutes another doctor phoned back and he said you, had better come down.* (117_1029 - Female patient in her 60s with chronic health issues, not in employment, caring responsibilities)

Confidentiality

A significant proportion of patients expressed concerns regarding confidentiality associated with the 'telephone-first' approach, as the system generally required the receptionist to ask the patient for brief details of their problem:

> *you know that whatever you say to a doctor is going to stay with the doctor, with the receptionist, you are never quite sure if it's going to stay there* (117_1066 - Single mother in her 30s, part-time work, infrequent user of GP)

Strong feelings were expressed on this subject, with one patient describing the approach as '*absolutely disgusting' (103_1042)* .Concern was even expressed by patients who acknowledged the benefit of providing the information in order for calls to be prioritized. Patients also reported concerns about confidentiality associated with the telephone consultation itself, especially if they had to receive the call back from the GP at a time and/or in a location where their conversation could be overheard, whether at home with family members present, in a work setting or on public transport.

Factors influencing whether the 'telephone-first' approach works for patients

As outlined above, there were differences between patients regarding the nature of the advantages and disadvantages reported in relation to the 'telephone-first' approach. It was also clear from the interviews that the value given to advantages and disadvantages varied significantly, even between patients from the same practice or with similar characteristics. For example, a disadvantage that represented a mild annoyance for one patient could represent a 'deal-breaker' for another, rendering the approach completely unacceptable. One patient described the effect of having to wait a long time for a call back from a GP whilst in a distressed state, and how this had influenced her decision to leave the practice:

For peer review only - http://bmjopen.bmj.com/site/about/guidelines.xhtml

> *I was really low and so I think I had to wait a few hours [for a call from the GP] and all that time I was in tears and it still took a couple of hours for the doctor. I thought, "Well, now I can't be bloody bothered."* (103_1042 – Female patient in her 50s, not in employment, mental and chronic physical health problems)

In addition, while there were common elements to the 'telephone first' approach, there was also significant variation between practices with respect to exactly how the approach was implemented and these could have a major effect on how patients responded to the new system. On the basis of our interview findings we identified characteristics of both practices and patients which influenced the acceptability of the 'telephone-first' approach to patients (tables 2 and 3). The way in which the 'telephone-first' approach was implemented and characteristics of the patients contributed to the acceptability of the approach to individual patients. The interplay between these two sets of factors was also important. Some patients provided long lists of annoyances (difficulty getting through on the telephone, confidentiality concerns when talking to receptionists, not being able to book in advance, not liking waiting for the call back) but still concluded that they preferred the new approach because they could speak to a doctor within hours and see them the same day if they needed to.

System / practice characteristic	Key features associated with acceptability to patients
**Capacity of the system to meet demand**	Telephone calls to the practice answered promptly
	Sufficient appointment slots available for both telephone and face-to-face appointments
**Flexibility of the approach**	Some advanced booking available
	Flexible timing offered for the GP to call back; ability to book the time of the call-back.
	Whether patients were required to describe their problem to the receptionist
	Adjustments for patients who found difficulty with the approach
**Capacity to preserve or enhance continuity of care**	Choice of GP offered for telephone consultation and subsequent face-to-face appointment
**Extent of patient education / knowledge**	Consultation with patients prior to introducing the approach
	Clear and updated instructions to patients on how the system works

Table 2. Practice characteristics which influenced the acceptability of the 'telephone-first' approach for patients.

For peer review only - http://bmjopen.bmj.com/site/about/guidelines.xhtml

Patient characteristic or resource	Key features likely to make the approach more acceptable to individual patients
**Communication skills**	Patients being articulate with good communication skills
**Confidence**	Patients having confidence to request the outcome they wanted
**Flexibility of daily schedule**	Patients able to accommodate time constraints of the approach e.g. being at home during the day/ retired/ working flexibly
**Access to mobile telephone**	Patients being easily accessible on a mobile telephone
**Value placed on face-to-face contact with GP**	Patients placing less value on face-to-face contact than on ease and speed of access to care
**Nature of relationship with GP or surgery**	Patients having a longstanding, trusting relationship with a GP and feeling comfortable communicating with him/her by telephone

Table 3. Patient characteristics which influenced the acceptability of the 'telephone-first' approach for individual patients.

Discussion

The study showed that, consistent with our published quantitative analysis of the patient and carer survey in our evaluation[2], patients expressed a wide range of views, often strongly held, on the 'telephone-first' approach. Qualitative interviews allowed us to understand these views in greater depth and to explore some of the reasons behind the different views expressed. The new system clearly suited some patients, (e.g. by allowing them to avoid coming into the surgery) but was problematic for others (e.g. when it was difficult for someone working in an open plan office to take a call-back). Variation was evident within as well as between the different patient groups we recruited from and appeared to be influenced by an interplay of individual and practice level characteristics. Notably, a substantial proportion of negative comments were about the operation of the scheme itself rather than the principles behind it, for example, difficulty getting through on the telephone or being unable to schedule when the GP would call back. Some practices were able to operate the scheme in a way that met their patients' needs better than others and practices appeared to vary significantly in how they had implemented the approach, according to patients' accounts.

The National Health Service in England has prioritized improving access to care for several years and the 'telephone first' approach is one attempt to address access problems, while at the same time trying to avoid an increase in practice workload. This study confirms previous research that there is considerable potential for using telephone consultations in general practice[5,10], and chimes with previous findings that access is not the main driver of patients' satisfaction with their GP practices – with interpersonal aspects of care and helpfulness of receptionists being more important.[11,12]

For peer review only - http://bmjopen.bmj.com/site/about/guidelines.xhtml

The study highlights the need for clinicians and policymakers to take the needs of patients with varying care-seeking and interaction approaches into account when making major changes to the organization of general practice care. This change, while designed to improve access to care and reduce the workload burden on practices, clearly did not meet the needs of all patients and provoked outright hostility in some, particularly among those who struggled to access care at all as a result of issues with how the scheme had been implemented. Practices considering making this change should reflect on how they can make the scheme flexible for patients' needs, how they can make it easy for patients to get through on the phone, and how they can use the approach to enhance both access and continuity of care, and recognize the need for continued development and adaptation of the approach.

A strength of the study is that the interviews included a wide range of patients and carers from a diverse group of practices, purposively sampled to capture a variety of views on the new approach. However, a limitation is the likelihood that practices operating the 'telephone-first' approach successfully were more likely to participate in the patient survey that provided patients who volunteered to be interviewed. We do not know how the views of patients participating in the study may compare with other patients, including those in practices that have not implemented the 'telephone-first' approach.

Questions that could be addressed by future research are how to develop systems that are flexible enough to meet the needs of all their patients. While a rigid 'telephone-first' approach for all consultations does not do this, we observed practices that were modifying this approach (by for example allowing for some advanced booking of appointments) often on an ongoing basis, to meet the needs of patients as closely as they could. Successful approaches are likely to be different in different practices and more work could be done to identify what works best in different circumstances and to share learning.

<u>Conclusions</u>

The 'telephone-first' approach appears to work well for some patients but others find it much less acceptable. Some of the reported problems related to how the approach had been implemented rather than the 'telephone-first' approach in principle and suggests there may be potential for some of the challenges to be overcome. A range of factors were identified that should be considered by practices planning the approach in order to maximise its acceptability and best meet the needs of patients.

Word count: 5178 words + 241 words in tables

<u>Acknowledgements:</u>

We would like to thank the patients and carers interviewed for this study and those who completed the questionnaires which provided the basis for participant recruitment. We are grateful to the GPs and staff at practices taking part in the study for their support with its conduct. We would also like to thank the GPs, practice manager and patients on the study steering group who gave guidance on the design and conduct of the study and all those who attended and contributed to study learning events.

For peer review only - http://bmjopen.bmj.com/site/about/guidelines.xhtml

Funding: The study was funded by the National Institute for Health Research (HS&DR Project 13/59/40). Part of the funding was used to pay for data to be extracted from practice records by one of the commercial companies providing management support for the 'telephone-first' approach (GP Access). GP Access had no input into the analysis or interpretation of the data. The study was sponsored by Cambridgeshire and Peterborough Clinical Commissioning Group (CCG), who gave initial approval for the project.

This article presents independent research funded by the National Institute for Health Research (NIHR). The views expressed are those of the author(s) and not necessarily those of the NHS, the NIHR or the Department of Health.

Competing interests: All authors have completed the ICMJE uniform disclosure form at www.icmje.org/coi_disclosure.pdf and declare: no financial relationships with any organisations that might have an interest in the submitted work in the previous three years; no other relationships or activities that could appear to have influenced the submitted work.

Author statement:

All authors contributed to the conception or design of the work, the interpretation of the findings. SB, JN, JC, JE were involved in data collection. SB, JN, JC, JE and EP conducted data analysis. All authors were involved in drafting and commenting on the paper and have approved the final version.

Ethical approval: The study was approved by the West of Scotland NHS Research Ethics Service (7th May 2015, REC reference 16/WS/0088).

Data sharing:

No additional data are available.

References

[1] Hobbs FD, Bankhead C, Mukhtar T, Stevens S, Perera-Salazar R, Holt T, et al. Clinical workload in UK primary care: a retrospective analysis of 100 million consultations in England, 2007-14. Lancet 2016;387:2323-30. https://doi.org/10.1016/s0140-6736(16)00620-6

[2] NHS England. High quality care now and for future generations: Transforming urgent and emergency care services in England. Page 35-36. https://www.england.nhs.uk/wp-content/uploads/2013/06/urg-emerg-care-ev-bse.pdf

[3] Newbould J, Abel G, Ball S, Corbett J, Elliott M, Exley J, Martin A, Saunders C, Wilson E, Winpenny E, Yang M, Roland M. Evaluation of telephone first approach to demand management in English general practice: observational study. BMJ 2017; 358: j4197 www.bmj.com/content/bmj/358/bmj.j4197.full.pdf

For peer review only - http://bmjopen.bmj.com/site/about/guidelines.xhtml

[4] Hallam L. Access to general practice and general practitioners by telephone: the patient's view. British Journal of General Practice 1993; 43: 331-335. www.ncbi.nlm.nih.gov/pmc/articles/PMC1372558/pdf/brjgenprac00041-0023.pdf

[5] McKinstry B, Watson P, Pinnock H, Heaney D, Sheikh A. Confidentiality and the telephone in family practice: a qualitative study of the views of patients, clinicians and administrative staff. Family Practice 2009; 26: 344-350 https://academic.oup.com/fampra/article/26/5/344/635397

[6] Brant, H, Atherton H, Ziebland S, McKinstry B, Campbell J, Salisbury C. Using alternatives to face-to-face consultations: a survey of prevalence and attitudes in general practice. British Journal of General Practice 2016; 66: e460-466. https://www.ncbi.nlm.nih.gov/pmc/articles/PMC4917048/

[7] Pereira Gray D, Wilkie P. Patient perspectives on the telephone first system. BMJ 2017; 359: j4925. https://doi.org/10.1136/bmj.j4925

[8] Boyatzis R. Transforming qualitative information: Thematic analysis and code development. Thousand Oaks, London, & New Delhi. 1998. SAGE Publications.

[9] O'Brien BC, Harris IB, Beckman TJ, Reed DA, Cook DA. Standards for reporting qualitative research: a synthesis of recommendations. Acad Med. 2014;89(9):1245-1251.

[10] Campbell J, Fletcher E, Britten N, Green C, Holt T, Lattimer V et al. Telephone triage for management of same-day consultation requests in general practice (the ESTEEM trial): a cluster-randomised controlled trial and cost-consequence analysis. Lancet 2014; 384: 1859-68. https://www.thelancet.com/pdfs/journals/lancet/PIIS0140-6736(14)61058-8.pdf

[11] Paddison CA, Abel GA, Roland MO, Elliott M, Lyratzopoulos G, Campbell J. Drivers of overall satisfaction with primary care: evidence from the English General Practice Patient Survey. Health Expectations 2013; 18: 1081–1092 https://onlinelibrary.wiley.com/doi/abs/10.1111/hex.12081

[12] Cowling T, Majeed A, Harris M. Importance of accessibility and opening hours to overall patient experience of general practice: analysis of repeated cross-sectional data from a national patient survey. British Journal of General Practice 2018; 18th June. http://bjgp.org/content/early/2018/06/18/bjgp18X697673?ct=

For peer review only - http://bmjopen.bmj.com/site/about/guidelines.xhtml

Appendix 1: Characteristics of participants and the 'telephone first' approach

Specific characteristics of the 'telephone first' approach (from patient perspective)	ID	Age[1]	Gender[1]	Ethnicity[1]	Health status[1]	Employment status, nature of health concerns and frequency of GP use[1 2]	Approach preference[2]
**Practice 100** (urban, list size 5,000-9,999)<br>**Notable features**: no advance booking of face-to face appointments; patient can specify time for call-back; nurse practitioner triages some requests; choice of GP offered for call-back and face-to-face appointment; duty GP takes phone calls in reception office<br>**Problems identified**: more difficult to see GP of choice on the day; can be difficult to get through to reception on Monday mornings<br>**Previous system:** ring up to book in advance or queue up for same day appointments; same-day appointments often not available	100_1004	71	Female	white British	fair	Retired; multiple chronic health issues; frequent user of GP	'Telephone first'
	100_1006	79	Male	white British	good	Retired; multiple chronic health issues, infrequent user of GP	'Telephone first'
	100_1064	Adult	Female	white British	fair	Carer for 85 year old mother with dementia; both have chronic health issues, frequent user of GP;	'Telephone first'
	100_1086	63	Male	white British	good	Recently retired; infrequent user of GP	'Telephone first'
**Practice 101** (urban, list size 5,000-9,999)<br>**Notable features**: possible to book telephone consultation in advance if preferred GP not available on the day; individual call back lists for each GP; prompt call-back or patient can specify time; some advance booking of face-to face appointments (for follow-ups or if patient unable to make same day appointment); nurse practitioner triages some requests<br>**Problems identified**: can sometimes be difficult to get through to reception<br>**Previous system:** ring up to book in advance; waited 2-3 days for appointment or longer for preferred GP	101_1002	76	Male	white British	very good	Retired; minor health issues requiring specialist input, infrequent user of GP; hearing impairment	'Telephone first'
	101_1006	65	Male	white British	very good	Full time carer for spouse; ongoing health issue requiring specialist input, infrequent user of GP	'Telephone first'
	101_1024	50	Female	other black	fair	Early retirement due to ill health; frequent user of GP	'Telephone first'
	101_1086	37	Male	white British	good	Works full time; ongoing mental and physical health issues; regular review by GP	'Telephone first'
**Practice 102** (urban, list size <5,000)<br>**Notable features**: quick response from reception to incoming calls; wait for call-back depends on urgency of the issue; some	102_1014	77	Female	white British	fair	Retired; multiple chronic conditions; frequent user of GP	'Telephone first'
	102_1019	67	Male	white British	poor	Retired; multiple chronic conditions; regular user of GP; seeing a specialist; lives alone	Conventional

advance booking of follow-up appointments; nurse does some telephone consulting; some forward booking by GPs, patient can always see GP face-to-face if they wish – practice considering making further modifications.<br>**Problems identified**: can sometimes be difficult to get through to reception but this is variable<br>**Previous system:** ring up to book in advance; often waited 3-4 days for appointment but same day appointments available when required	102_1031	47	Female	white British	poor	Works part time; ongoing mental and physical health issues; frequent user of GP; hearing impairment	'Telephone first'
	102_1064	65	Female	white British	good	Retired; infrequent user of GP	'Telephone first'
**Practice 103** (urban, list size 5,000-9,999)<br>**Notable features**: receptionist asks patient whether issue is urgent – call backs prioritised dependent on urgency of issue; flexibility in scheduling call back – patient can request a call back on another day if preferred GP is not in; no advance booking of face-to-face appointments<br>**Problems identified**: can be difficult to get through to reception – phone line sometimes goes dead; face-to-face appointments not available if call later in the day requiring patient to call again the following day<br>**Previous system:** walk-in system for on the day appointments or book by phone – 2/3 days wait	103_1030	41	Female	white British	fair	Mother of two disabled children; frequent user of GP often for advice by phone	'Telephone first'
	103_1034	78	Male	white British	fair	Retired; very frequent user of GP	'Telephone first'
	103_1042	50	Female	white British	no response	Does not work; mental and chronic physical health problems; frequent user of GP	Conventional
	103_1053	71	Female	white British	good	Retired; frequent user of GP	'Telephone first'
	103_1074	67	Female	white British	fair	Retired; infrequent user of GP	'Telephone first', though with modifications
**Practice 104** (urban, list size <5,000)<br>**Notable features**: receptionist asks patient for a reason for the call -GP reviews list of reasons given and offers face-to-face appointments to some patients on basis of this information alone (without speaking to patient directly); call-back within an hour by GP or by receptionist to call in for a face-to-face appointment<br>**Problems identified**: can be difficult to get through to reception on the phone on a Monday	104_1070	54	Female	white British	fair	Does not work due to chronic health problems; infrequent user of GP as condition well controlled	'Telephone first'
	104_1087	74	Female	white British	good	Retired; increasing frequency of GP visits with age	'Telephone first'

For peer review only - http://bmjopen.bmj.com/site/about/guidelines.xhtml

**Previous system:** walk-in system							
**Practice 105** (urban, list size ≥ 10,000)<br>**Notable features**: call-back within 30 minutes for urgent issues (wait for call-back depends on urgency); cut off time for patients to call by in order to receive same day call back (e.g. 16.30); nurse triage for some requests; choice of GP offered for call-back and face-to-face appointment; reception spread calls across all GPs, set number of calls per GP per day then a pooled list.<br>**Problems identified**: can be difficult to get through to reception on the phone; online booking no longer available<br>**Previous system:** booking in advance by phone – no difficulty getting an appointment but up to three week wait for non-emergency appointment; on line booking facility	105_1040	79	Female	white British	good	Retired; chronic health issues; frequent user of GP; hearing impairment	'Telephone first'
	105_1043	Adult[3]	Female	white British	n/a	Does not work – mother of young child; chronic health issues (self and child); frequent user of GP	'Telephone first'
	105_1090	78	Male	white British	fair	Retired; multiple chronic health issues; frequent user of GP	'Telephone first'
	105_1099	78	Male	white British	very good	Retired; fit and active; infrequent user of GP	'Telephone first'
**Practice 106** (urban, list size ≥ 10,000)<br>**Notable features**: variable wait for call-back (from almost instant to many hours); choice of GP offered for call-back and face-to-face appointment; some flexibility for GP to book appointment for next day but no advance booking by reception (e.g. follow-up appointments); patients can choose time for call back.<br>**Problems identified**: can be difficult to get through to reception on the phone on a Monday; can wait all day for a call back<br>**Previous system:** booking in advance by phone – was beginning to get more difficult to get an appointment	106_1013	53	Female	white British	nr	Works flexibly from home; chronic health issue; anxiety; frequent user of GP; previous missed cancer diagnosis	Conventional
	106_1025	78	Female	white British	fair	Retired; chronic health issue; frequent user of GP	'Telephone first'
	106_1026	45	Female	white British	good	Not currently working due to ill health; infrequent user of GP	'Telephone first'
	106_1064	68	Female	white British	fair	Retired; chronic health issues but infrequent user of GP	'Telephone first'
	106_1077	61	Female	white British	fair	Does not work; mental health and multiple chronic physical health problems; frequent user of GP	'Telephone first'
**Practice 108** (urban, list size <5,000)<br>**Notable features**: variable wait for call-back (from 30 minutes to many hours); duty GP takes calls all day, others only 8-11am; no advance bookings; recorded message indicates cut off time after which only emergency cases will receive a call back (e.g. 15.00)<br>**Problems identified**: variable reports regarding difficulty getting	108_1032	59	Female	white British	good	Works full time but easy to take calls or make appointments; chronic condition; carer for elderly parents (with hearing impairment); frequent user of GP for self and as carer;	'Telephone first'
	108_1090	66	Female	white British	good	Retired; infrequent user of GP	Conventional
	108_1099	28	Female	Chinese	good	Student – some difficulty taking calls or	N/a (only

through on the phone; no longer offered choice of preferred GP; can wait all day for a call back; same-day call back not always available<br>**Previous system:** booking in advance by phone – was beginning to get more difficult to get an appointment						making appointments; speaks English as a second language; unfamiliar with UK health system; frequent contact with GP	experienced this system)
**Practice 110** (urban, list size 5,000 – 9,999)<br>**Notable features**: phone lines shut off early in the day with recorded message to call the following day; no advance booking available; time of call-back not indicated; separate walk in system also reported to be in operation (bypassing phone system)<br>**Problems identified**: extreme difficulty getting through on the phone; if patient gets through appointments are often unavailable and patient is asked to call the following day; no longer offered choice of preferred GP; can wait all day for a call back;<br>**Previous system:** Advance booking system with long wait of a week or sit and wait on the day. Previously had online system but scrapped.	110_1007	60	Female	white British	fair	Early retirement due to ill health; frequent user of GP	Conventional
	110_1026	74	Male	white British	poor	Retired; multiple chronic conditions requiring specialist input; mental health issues; lives alone; reports limited user of GP due to Telephone	Conventional
	110_1095	63	Female	white/black	fair	Part time/voluntary work; ongoing mental health issues; reports limited user of GP due to Telephone	Conventional
**Practice 112** (urban, list size 5,000 – 9,999)<br>**Notable features**: receptionist asks for brief details of issue – patient either put straight through to GP or receives very prompt call back; no advance booking available; separate system for nurse appointments<br>**Problems identified**: difficulty getting through on the phone – might take up to an hour; if patient calls after 9 am call backs are often unavailable and patient asked to call the following day; long wait in the surgery for booked appointment<br>**Previous system:** turn up at 8:00am and sit and wait on the day.	112_1015	65	Female	white British	good	Retired; infrequent user of the GP	'Telephone first'
	112_1046	Adult[3]	Male	other	n/a	Working parent,; speaks English as a second language	'Telephone first'
**Practice 114** (urban, list size 5,000 – 9,999)	114_1008	48	Male	white British	good	Works/easy to take calls or make	'Telephone first'

For peer review only - http://bmjopen.bmj.com/site/about/guidelines.xhtml

**Notable features**: receptionist does not ask about the nature of the issue (change from original system); receptionist provides indication of time for call-back and can schedule flexibly around patient's requirements; advance booking available for some follow-up appointments; nurse practitioner does some telephone consulting<br>**Problems identified**: system functioning well<br>**Previous system**: ring to book face-to-face appointment same day appointments were always available if required.						appointments; chronic health issues; frequent user of GP	
	114_1029	Adult[3]	Female	white British	n/a	Carer for elderly father; works from home; frequent user of GP for self and as carer	'Telephone first'
	114_1058	72	Female	white British	poor	Retired; chronic health issues; frequent user of GP	Conventional
**Practice 117** (urban, list size 5,000 – 9,999)<br>**Notable features**: prompt call-back from GP (often within 10-15 minutes – maximum 1 hour 30 minutes); no advance booking of face-to-face appointments; if preferred GP is not available patient offered choice to speak to a different GP or ring back when available; call back only available for emergencies after 16.00<br>**Problems identified**: time cut off to ensure face-to-face appointment available on the day is unclear;<br>**Previous system**: same day appointment system - rang on the day and had to see whoever was available that day or ring the next day. Sometimes a long wait to see Dr of choice	117_1027	51	Female	white British	very good	Works/difficult to take calls; infrequent user of GP;	'Telephone first'
	117_1029	60	Female	white British	poor	Does not work due to ill health and caring responsibilities; multiple chronic conditions; very frequent user of GP	'Telephone first'
	117_1066	32	Female	white British	good	Single mother/part time voluntary work; infrequent user of GP	'Telephone first'
	117_1073	86	Male	white British	good	Retired; recent hospital stay but previously in good health; infrequent user of GP	'Telephone first'

peer review only

Appendix 2. Patient interview guide

This was a broad guide which was developed iteratively as early findings were reviewed by members of the research team

Introduction:

Please can you tell me a little bit about yourself, including whether you work

How long have you been at the practice?

How is your health in general? How regularly do you need access the GP?

Process of booking an appointment:

How do you make an appointment with your GP?

Has this changed since you have been a patient at the practice? (What used to happen?)

Have you had to call the surgery on behalf of someone else / or has someone else called on your behalf? How did that work?

When you call the surgery, how long does it take to get through? Have you experienced any difficulty in getting through?

Do you have to provide any information to the reception staff concerning the call? [could prompt here for how comfortable they feel speaking to receptionist?]

If offered a call back do you get it within a reasonable time period? Do you get to specify when you would like the call back? Have you had any difficulties around the call back?

Is it always a doctor that calls you back? If you have a preferred doctor, do you get to speak to them? [prompt on continuity of care]

Have there been any changes in how the telephone consultations have been run since they were introduced? Has that improved the service/ made it more difficult for you?

Your experience with telephone consultations:

How have you found the experience of using this system?

Is there anything about your lifestyle that makes it more or less difficult for you to use? [prompt: working hours, caring responsibilities etc]

What was the outcome of your last telephone consultation with the GP? (led to a face-to-face appointment, directed to another health-care professional such as nurse, or another service eg. Social services etc. or just given advice over the phone)

> *If not offered a face-to face appointment with GP:*
>
> Were you happy with this outcome? Do you feel the service was satisfactory? Did you seek care elsewhere instead? (e.g. at A&E)

For peer review only

If offered a face-to-face appointment:

If you have a preferred doctor, did you get to see him/her?

Have you noticed any change in the actual appointment [get more time with GP, GP better prepared etc]? Has the quality of the appointment changed?

Have you had other telephone consultations where something different happened? What was the outcome? [repeat prompts as above])

How you feel about the system and what it means for you:

What do you like about the current system for making appointments? ([prompt on convenience, chance to speak to GP when don't want appointment – reassurance, practice seems more organised, get an appointment when want one, appointments not being booked by people for unsuitable use]

What do you dislike about the current system for making appointments? [don't get an appointment when want one, can't communicate well using phone – lost personal connection, elderly – loss of social contact, safety concerns]

Is there anything you miss about the old system (e.g. being able to plan in advance)?

How do you feel about talking to the doctor on the phone? [prompts:Do you feel comfortable talking about your medical condition /do you have any concerns over confidentiality? Do you feel that you are able to make yourself understood?]

How did you feel about the system when it was first introduced? Has this changed over time? [i.e. have they got used to the system]

Has the telephone appointment system changed the way you seek health care services? [prompts: are they thinking about alternative services more before contacting GP? i.e. is this right for the GP should I be going to nurse or don't bother go straight to A&E?]

Has your contact with the GP surgery changed? (i.e. ring more often as know can speak to doctor etc). [prompt on whether offered an appointment, if not with GP who with? Just given advice over phone etc]

Concluding:

If you had the choice would you go back the old system or keep the new system? Why would you make that choice?

Do you think other patients share your view?

Overall are you happy with the care you receive at the practice?

Is there anything else about the telephone appointment system that you'd like to discuss that we haven't spoken about?

Thank you for taking the time to meet with me today

For peer review only

Standards for Reporting Qualitative Research (SRQR)

<u>Title and abstract</u>
S1 Title Concise description of the nature and topic of the study Identifying the study as qualitative or indicating the approach (e.g., ethnography, grounded theory) or data collection methods (e.g., interview, focus group) is recommended

The nature / topic of the study is included.

S2 Abstract Summary of key elements of the study using the abstract format of the intended publication; typically includes background, purpose, methods, results, and conclusions

These are all included in the abstract.

<u>Introduction</u>
S3 Problem formulation Description and significance of the problem/phenomenon studied; review of relevant theory and empirical work; problem statement

The nature and significance of the problem is described along with a review of empirical work.

S4 Purpose or research question Purpose of the study and specific objectives or questions

The objectives of the study are stated in the last sentence of the introduction.

<u>Methods</u>
S5 Qualitative approach and research paradigm Qualitative approach (e.g., ethnography, grounded theory, case study, phenomenology, narrative research) and guiding theory if appropriate; identifying the research paradigm (e.g., postpositivist, constructivist/ interpretivist) is also recommended; rationale (The rationale should briefly discuss the justification for choosing that theory, approach, method, or technique rather than other options available, the assumptions and limitations implicit in those choices, and how those choices influence study conclusions and transferability. As appropriate, the rationale for several items might be discussed together).

See response S6 below

S6 Researcher characteristics and reflexivity Researchers' characteristics that may influence the research, including personal attributes, qualifications/experience, relationship with participants, assumptions, and/or presuppositions; potential or actual interaction between researchers' characteristics and the research questions, approach, methods, results, and/or transferability

The approach was principally narrative led by a flexible interview guide which is reproduced in appendix 1. However, as we outline in the paper, the topic guide was developed iteratively, both allowing flexibility within the interview for patients to express their own concerns and to introduce elements which had arisen from preliminary analysis of earlier interviews.

S7 Context Setting/site and salient contextual factors; rationale

These are described in the methods and results.

For peer review only

S8 Sampling strategy How and why research participants, documents, or events were selected; criteria for deciding when no further sampling was necessary (e.g., sampling saturation); rationale

The sampling method is described in the method section, including possible biases that may have been introduced by the method of selecting practices.

S9 Ethical issues pertaining to human subjects Documentation of approval by an appropriate ethics review board and participant consent, or explanation for lack thereof; other confidentiality and data security issues

This study was reviewed and given a favourable opinion by the West of Scotland Research Ethics Committee 5 (reference: 15/WS/0088).

S10 Data collection methods Types of data collected; details of data collection procedures including (as appropriate) start and stop dates of data collection and analysis, iterative process, triangulation of sources/methods, and modification of procedures in response to evolving study findings; rationale

These are included in the method section

S11 Data collection instruments and technologies Description of instruments (e.g., interview guides, questionnaires) and devices (e.g., audio recorders) used for data collection; if/how the instrument(s) changed over the course of the study

These are described in the methods section

S12 Units of study Number and relevant characteristics of participants, documents, or events included in the study; level of participation

These are described in the results with further detail provided in a supplementary file

S13 Data processing Methods for processing data prior to and during analysis, including transcription, data entry, data management and security, verification of data integrity, data coding, and anonymization/deidentification of excerpts

These are described in the methods section

S14 Data analysis Process by which inferences, themes, etc., were identified and developed, including the researchers involved in data analysis; usually references a specific paradigm or approach; rationale

This is described in the method section, including the iterative development of the interview guide and the procedure for checking coding.

S15 Techniques to enhance trustworthiness Techniques to enhance trustworthiness and credibility of data analysis (e.g., member checking, audit trail, triangulation); rationale

This is described in the method section (multiple coding, regular discussion of emerging findings in the research team).

For peer review only

Results/findings

S16 Synthesis and interpretation Main findings (e.g., interpretations, inferences, and themes); might include development of a theory or model, or integration with prior research or theory

The results section includes both the main findings and a synthesis of factors affecting the acceptability of the telephone-first approach.

S17 Links to empirical data Evidence (e.g., quotes, field notes, text excerpts, photographs) to substantiate analytic findings

The results section contains quotations from patient and carer interviews to substantiate the findings

Discussion

S18 Integration with prior work, implications, transferability, and contribution(s) to the field. Short summary of main findings; explanation of how findings and conclusions connect to, support, elaborate on, or challenge conclusions of earlier scholarship; discussion of scope of application/ generalizability; identification of unique contribution(s) to scholarship in a discipline or field

These are included in the discussion.

S19 Limitations Trustworthiness and limitations of findings

These are included in the discussion and also in the bullets which follow the abstract.

S20 Conflicts of interest Potential sources of influence or perceived influence on study conduct and conclusions; how these were managed

A conflict of interest statement has been completed by all authors.

S21 Funding Sources of funding and other support; role of funders in data collection, interpretation, and reporting

The source of funding and the role of funders is included.

For peer review only

BMJ Open

A qualitative study of patient views on a 'telephone-first' approach in general practice in England: speaking to the GP by telephone before making face-to-face appointments

Journal:	*BMJ Open*
Manuscript ID	bmjopen-2018-026197.R1
Article Type:	Research
Date Submitted by the Author:	09-Nov-2018
Complete List of Authors:	Ball, Sarah L.; RAND Europe, Cambridge Centre for Health Services Research<br>Newbould, Jennifer ; RAND Europe<br>Corbett, Jennie; RAND Europe<br>Exley, Josephine; London School of Hygiene and Tropical Medicine<br>Pitchforth, Emma; RAND Europe,<br>Roland, Martin; University of Cambridge, GP and Primary Care Research Unit
**Primary Subject Heading**:	General practice / Family practice
Secondary Subject Heading:	Qualitative research
Keywords:	General practice, Remote consultation, Appointments and schedules, Telephone, Patient satisfaction

SCHOLARONE™
Manuscripts

A qualitative study of patient views on a 'telephone-first' approach in general practice in England: speaking to the GP by telephone before making face-to-face appointments

Sarah Ball [1], Jennifer Newbould[1], Jennie Corbett[1], Josephine Exley[1], Emma Pitchforth[1] [3], Martin Roland[2].

[1] Cambridge Centre for Health Services Research, RAND Europe, Westbrook Centre, Cambridge CB4 1YG, UK

[2] Cambridge Centre for Health Services Research, Institute of Public Health, University of Cambridge, Forvie Site, Cambridge CB2 0SR, UK

[3] University of Exeter University of Exeter Medical School, St Luke's Campus Heavitree Road, Exeter EX1 2LU, UK

Correspondence to: Sarah Ball, Cambridge Centre for Health Services Research, RAND Europe, Westbrook Centre, Cambridge CB4 1YG, UK, Email - sarahb@rand.org, Telephone - +44 1223 353 329 x 2594

Author email addresses:

Jennifer Newbould - newbould@rand.org

Jennie Corbett - jenniecorb@gmail.com

Josephine Exley - Josephine.Exley@lshtm.ac.uk

Emma Pitchforth - E.Pitchforth@exeter.ac.uk

Martin Roland - mr108@medschl.cam.ac.uk

For peer review only

Abstract

Objective: To understand patients' views on a 'telephone-first' approach, in which all appointment requests in general practice are followed by a telephone call from the GP.

Design: Qualitative interviews with patients and carers

Setting: Twelve general practices in England

Participants: 43 patients, including 30 women, nine aged over 75, four parents of young children, five carers, five patients with hearing impairment and two whose first language was not English.

Results: Patients expressed varied views, often strongly held, ranging from enthusiasm for to hostility towards, the 'telephone-first' approach. The new system suited some patients, avoiding the

For peer review only - http://bmjopen.bmj.com/site/about/guidelines.xhtml

need to come into the surgery, but was problematic for others e.g. when it was difficult for someone working in an open plan office to take a call-back. A substantial proportion of negative comments were about the operation of the scheme itself rather than the principles behind it, for example difficulty getting through on the phone or being unable to schedule when the GP would phone back. Some practices were able to operate the scheme in a way that met their patients' needs better than others and practices varied significantly in how they had implemented the approach.

Conclusions: The 'telephone-first' approach appears to work well for some patients but others find it much less acceptable. Some of the reported problems related to how the approach had been implemented rather than the 'telephone-first' approach in principle and suggests there may be potential for some of the challenges experienced by patients to be overcome.

247 words

Key words

General practice

Remote consultation

Appointments and schedules

Telephone

Patient satisfaction

Strengths and limitations of this study

Participants included a wide range of patients and carers from a diverse group of practices.

- Patients and carers selected for interview had recent experience of the 'telephone-first' approach.
- Participants were purposively sampled to include a wide range of views on the new approach.
- Semi-structured interviews allowed participants to discuss in detail their own experiences of the 'telephone-first' approach
- Practices agreeing to take part in the study may have been operating the 'telephone-first' approach more successfully than those that declined.

Introduction

Increasing demand for general practice care is leaving practices in the UK struggling to meet patient need.[1] In response, some practices (at least 150 in England) have adopted a novel 'telephone-first' approach to managing patient requests for a consultation. In this whole system approach, all appointment requests are followed by a telephone call from the general practitioner (GP). Either the issue is resolved during this call through provision of advice, a prescription or redirection to another health professional, or the patient is invited for a face-to-face consultation, usually on the same day.

Currently, two commercial companies (Dr First and GP Access) promote this approach in the UK and provide management support to practices adopting it. The approach has been advocated by NHS

For peer review only - http://bmjopen.bmj.com/site/about/guidelines.xhtml

England based on significant benefits reported by the companies including improved access to primary care, improved patient satisfaction and reductions in both primary and secondary care utilisation.[2] However, an independent evaluation that we carried out found no evidence of an overall reduction in GP workload, no evidence of reduced secondary care costs and, while patients were able to be seen much more quickly, there was little overall improvement in patient satisfaction as expressed in patient surveys.[3]

While published studies on patient satisfaction with GP telephone consultations in general report positive findings[4,5,6], the 'telephone-first' approach is a much more fundamental innovation in service provision and the National Association for Patient Participation has raised a range of concerns and opposition to the approach.[7] The patient surveys described in a previous paper [3] elicited a wide range of views about the telephone-first approach, from strongly positive to strongly negative. In this paper, we report the findings of qualitative interviews conducted with patients and carers to explore these views in greater depth.

Methods

Site selection, sampling and recruitment

Qualitative interviews with patients were undertaken in twelve GP practices using the telephone-first approach. Participating practices came from areas of England including the North East, North West, Midlands, East Anglia, London, the South East and the South West. Practices were selected purposively from the twenty practices participating in a patient and carer survey as part of our wider evaluation,[2] to include those with a range of experiences of adopting the 'telephone-first' approach, including practices reporting positive experiences and those that had experienced or overcome problems.

In the first instance, patients who were potential participants indicated their interest in being contacted for an interview by returning a reply slip that accompanied the patient and carer survey. Purposive sampling of those who expressed an interest was carried out by the research team, to gain a range of views and to ensure people with the following characteristics were included: older people, people who work, people with disabilities, people with chronic conditions and those with English as a second language. Selected interested participants were contacted by a member of the research team by the preferred contact mode indicated in the reply slip (telephone or email) and invited to take part in an interview.

Data collection

Semi-structured interviews were conducted by four researchers (SB, JN, JC, JE), either at the patient's home or at their GP surgery, as requested by the patient. All interviewees gave written consent to be interviewed. A common interview guide, informed by the literature, was used for each interview (see Appendix 1), although emphasis was given to allowing participants to talk from their own perspective and elements of the guide were developed iteratively as the study progressed. The main focus of the interview was on patients' and carers' views of the advantages and disadvantages

For peer review only - http://bmjopen.bmj.com/site/about/guidelines.xhtml

of the ‘telephone-first’ approach including its convenience, perceptions of quality of care and impacts on the doctor-patient relationship. Interviews were audio-recorded with the participants’ permission, and transcribed verbatim. Transcripts were anonymized by removing references to identifiable names and places.

Data analysis and reporting

Data analysis proceeded in parallel with data collection and informed the iterative development of the interview topic guide. Thematic analysis of the data was conducted based on the principles outlined by Boyatzis.[8] Transcripts were read and re-read and ‘codes’ applied to meaningful sections of text. Coding was conducted by SB, JC, JE, JN and EP. As analysis progressed codes were grouped into overarching or organising themes using NVivo 10 software. Data within themes were scrutinized for confirming and disconfirming views across the range of participants. Emerging findings were shared and discussed regularly within the study team. We have followed SRQR reporting guidelines.[9]

Patient involvement

A study steering group was established, which included four patients along with healthcare professionals. The steering group met on three occasions and provided input into the design and conduct of the study including advice on patient materials produced during the study. Patient representatives from the steering group and those from participating practices attended a learning event at which practices shared their experiences of the ’telephone-first’ approach and commented on our findings to inform their interpretation.

Results

Interviews were conducted with 43 patients and carers registered at 12 GP practices across England, all of which had been using the ‘telephone-first’ approach for between 18 months and 5 years. Respondents were aged between 28 and 86 years and included older people, parents of young children, carers, working people and a number of other ‘hard to reach’ groups (Table 1).

The practices at which the patients were registered varied with respect to: list size, geographical location and a range of characteristics of the catchment population, such as deprivation and ethnicity. While there were common elements to the ‘telephone-first’ approach used across the practices under study, there was also significant variation with respect to exactly how the approach was implemented. The characteristics of the practices, the specifics of the ‘telephone-first’ approach used within them and further details on the characteristics of the patients and carers interviewed are outlined in Appendix 2.

Characteristics	Number of interview participants (%)
**Female**	30 (69.8%)

For peer review only - http://bmjopen.bmj.com/site/about/guidelines.xhtml

**Aged over 75**	9 (20.9%)
**Parent of child under 13 years**	4 (9.3%)
**Carer[1]**	5 (11.6%)
**Working**	11 (25.6%)
**Hearing impaired**	5 (11.6%)
**First language not English**	2 (4.7%)
**Living with a chronic condition**	24 (55.8%)
Total	**43**

[1] All five carers were interviewed in both their capacity as a carer and as a patient

Table 1. Characteristics of interview participants

Interviews provided a rich source of data, and patients and carers were open in expressing their views (whether enthusiastic, ambivalent or hostile towards the approach). Whilst the majority of patients, when asked to make a choice, said that they would stick with the 'telephone-first' approach rather than return to the system that their practice had run previously, responses were nonetheless extremely varied: some patients reported being highly satisfied (giving strong endorsements), while others found the approach unacceptable. In describing their experiences of the approach, patients outlined a broad range of advantages and disadvantages in relation to its impact on how they were able to access care and the nature or quality of the care received. A number of themes arose in the analysis, which we present below.

Impact on initial contact with the practice

A clear theme was the impact of the 'telephone-first' approach on the nature of the initial contact made with the practice when booking an appointment. The perceived impact varied, with some patients describing how the initial contact was more streamlined following the changes, while others reported difficulties with or objections to the new booking process (such as long waits for calls to be answered or restricted opening times for telephone lines). For example, one patient, among a number who reacted with hostility to the introduction of the approach, described a situation in which it had taken days to get through to the practice to make an appointment:

> *…. tried for two days, press five [for automatic redial] still off - and on the Thursday someone actually answered. […] Said 'what is it?' so I said what [was wrong] and I need to see the Doctor. They phoned me back then. She says well Doctor [name redacted] is not in today - phone tomorrow. Bump [phone being hung up]. So I phoned the next morning 8 o'clock. Phones off. I phoned every five minutes till 8.30am - it came on, 'surgery's now full', phone Monday. […]You should try the system… It's that bad you couldn't make it up. If they had someone to report it to I'd prosecute them. They're terrible.* (110_1026 – Male patient in his 70s, retired, multiple chronic conditions and mental health issues)

For peer review only - http://bmjopen.bmj.com/site/about/guidelines.xhtml

Patients attributed difficulties getting through to the practice to the way the approach had been implemented (such as a lack of reception staff to answer the telephone or shutting the phone lines early), but also highlighted that the issues caused particular problems for them as individuals, for reasons such as lacking the time to wait to get through on the telephone, or difficulty calling at the required time of day (particular issues for working people), or as a result of a personal preference for making the initial contact with the practice in person rather than by telephone.

> *I just don't like it [the 'telephone-first' approach]. […] I just want a doctor's where I can go in, phone up, whatever which way I want to do it, book an appointment and go.* (103_1042 – Female patient in her 50s, not in employment, mental and chronic physical health problems)

Responsiveness of the practice to patient needs

A further theme related to the perceived impact of the 'telephone-first' approach on the degree to which the practice was able to be responsive to patient needs. Patients at some practices commented positively on the prompt response of GPs following their initial call to the surgery. Guaranteed same-day call-backs (in some cases within minutes or within an agreed time slot) reassured patients who were anxious about what might be wrong with them, and the availability of timely face-to-face appointments (if required) was appreciated:

> *This way I find if he [the GP] deems it serious enough for you to call in to see him, he'll see you the same day, which is brilliant.* (100_1004 – Female patient in her early 70s, retired, multiple chronic health issues)

> *Apart from just jumping in the car and going walking into a doctor's, there's no other way you could improve that.* (101_1002 – Male patient in his 70s, retired, minor health issues requiring specialist input, hearing impairment)

Patients at some practices however, described a delayed or unpredictable response, with no indication as to when the doctor would call back, or a lack of availability of appointments after speaking to the doctor. Variability between the reports of patients registered at different practices indicated that there were variations in the way the call-back system was managed, or in the capacity of practices to adequately meet demand (with respect to the availability of sufficient appointment slots for both telephone and face-to-face appointments).

In addition, interviewees described how their own personal characteristics or circumstances meant that they found unpredictability (with respect to receiving a response from the practice) particularly difficult, including a patient whose job as a support worker meant that she was unable access her mobile phone during a shift and patients with mental health issues who reported feeling anxious or distressed while they waited for a response from the GP. There was some acknowledgement among patients commenting on the inconvenience of having to wait for a call back, that this had not been a particular issue for them, but indicated concern that it would be an issue in case of an urgent need.

Implications for equitable / fair access to care

For peer review only - http://bmjopen.bmj.com/site/about/guidelines.xhtml

Patients were aware of and expressed strong views with respect to the implications of the ‘telephone-first’ approach for fairness and equity in access to care. Some patients interviewed indicated that they appreciated that the ‘telephone-first’ approach led to more efficient use of resources and improved access for patients with the greatest need for urgent care, and recognized that this in turn conferred benefits to them as individuals (ensuring prompt access if required):

> *you get to speak to a doctor before you go in for your appointment, because I think there are a lot of times when you actually don’t need to see a doctor face to face but, sometimes the advice of a doctor can put your mind at ease or just give you the information that you need to know - so then, you are not wasting your time and you are not wasting their time.* (117_1066 – Female patient in her 30s, single mother in part time work, infrequent user of GP)

> *It’s better this way because then you don’t get any timewasters. […] Then you haven’t got to wait. They put you first before the timewasters.* (105_1043 - Female patient, mother of young child, both with chronic health issues)

Patients differed in their perceptions of the intended function of the approach with respect to redirecting patient demand. While some patients perceived the ‘telephone-first’ approach to be a fair system for meeting patient need, others saw it as a barrier, intended to keep as many patients as possible away from face-to-face appointments with busy doctors, describing feeling the need to *‘fight’* or *‘protest’* to justify their requests to see a doctor face-to-face:

> *It certainly feels like a gate-keeping service […] like being kept as much at arm’s length as possible.* (114_1058 –Female patient in her early 70s, retired, chronic health issues)

Some expressed concern on behalf of vulnerable patients, such as the elderly or those with mental health issues, who may lack the confidence or communication skills to push for an appointment when required.

Ease and convenience of access to care

A strong theme in the analysis centred on how the introduction of the ‘telephone-first’ approach had affected the ease and convenience with which patients were able to access care. For some, the new mode of access had resulted in increased convenience, while for others the opposite had been the case. Commonly, the patients interviewed reported that they found that the approach enabled more convenient access to advice and care than the system previously in place, with benefits including: being able to get on with daily activities while waiting for a response from the practice, rather than having to wait for long periods in the surgery (facilitated by the availability of mobile telephone contact); reduced need to travel to the surgery unnecessarily (a particular benefit for those for whom travelling to the surgery was difficult, such as a mother with disabled children, a carer whose husband was disabled with chronic conditions and mobility issues, and those dependent on limited public transport services); and access to medication without the need for a face-to-face appointment:

> *I like the fact that on a day like today, it is chucking it down, it’s miserable, it’s cold, if my mum had had to come to the doctor instead of a phone call on*

For peer review only - http://bmjopen.bmj.com/site/about/guidelines.xhtml

> *any day where the weather was like this, it would have caused her a lot of pain.* (102_1031 – Female patient in her 40s, works part time, ongoing mental and physical health issues)

In particular, patients able to accommodate time constraints of the approach (e.g. being at home during the day, retired or working flexibly) highlighted how the 'telephone-first' approach fitted conveniently with their daily schedules. Others, however, found the approach inconvenient in one or more respects, including: not being able to book appointments in advance; receiving a call from the GP at inconvenient times (when shopping, on public transport, or at work); or having to stay at home to wait for a call, particularly if it related to a personal issue that it was difficult to discuss in public:

> *you can't sit glued to your phone all day waiting for a call, even if you've got a mobile phone, you might be in the shower, or you might be in a shop or on the other phone or something. So it doesn't work… and how people who are working, expect to get an appointment I don't know really.* (110_1007 – Female patient, early retirement due to ill health)

Patients at several practices, described how such issues had been addressed by their practice by ensuring flexibility in the approach, such as by accommodating patient requests for a call-back at a particular time or offering limited advanced bookings for those unable to attend on the same day.

Similarly, patients with particular difficulties that had an impact on how they were able to interact with the practice using the 'telephone-first' approach, described how such difficulties were overcome by minor adjustments and a flexible approach e.g. special arrangements for patients whose first language was not English, or those with a hearing impairment.

Differences in the nature of GP consultations: efficiency, communication and social contact

Patients highlighted differences in the nature of GP consultations as a result of the new approach, not only identifying differences between telephone and face-to-face consultations, but also the impact of initial telephone contact on subsequent face-to-face appointments. Patients described both advantages and disadvantages of initially consulting by telephone rather than face-to-face, reflecting individual differences with respect to confidence and efficacy of communication by telephone and the value placed on face-to-face contact with a GP.

While some patients described feeling very comfortable communicating by telephone, including some patients with mental health issues who preferred telephone consultations because they felt more relaxed, others reported difficulties describing symptoms or understanding and recalling the GP's advice. Those patients reporting that they felt anxious when communicating on the telephone included older people, those with mental health issues, hearing impairment and one for whom English was not his first language. Others reported concerns on behalf of other patients:

> *I've got a friend, an old lady who's 88, going on 89, I think, and she absolutely hates [it]. She says "I can't talk on the phone, I just don't know what to say, I just go to pieces." And somebody like her, it's just totally awful you know, it's not satisfactory at all.* (110_1007 – Female patient in her 60s, early retirement due to ill health)

For peer review only - http://bmjopen.bmj.com/site/about/guidelines.xhtml

Some interviewees commented that they found the approach to be impersonal – rushed and to the point, an issue that was highlighted by patients with mental health concerns and chronic conditions in particular. This was in part attributed to a lack of relational continuity of care (see below) but also due to the nature of the telephone consultation itself, and the absence of the social cues present in face-to-face interaction. A patient with mental health issues described the negative impact of a lack of face-to-face contact on the nature of the consultation:

> *I just cannot cope with not seeing someone's face […] I just want to speak in a room with the door closed face-to-face with someone so that I can be honest about how I am feeling and what's been happening lately. So I don't really say much over the phone […] whereas if it was face-to-face I would explain more* (110_1095 – Female patient in her 60s, part-time work, ongoing mental health issues)

Changes in the nature of face-to-face appointments following the introduction of the 'telephone-first' approach, were also noted, including improvements such as reduced waiting time in surgery and a calmer more relaxed atmosphere, with patients experiencing less time-pressure during appointments. Some patients suggested that the approach led to GPs being better prepared and the appointment being more streamlined as a result. A few among those who did not observe any difference in the nature of face-to-face appointments, however, commented that having to repeat details given over the telephone in the face-to-face appointment was an annoyance and appeared inefficient.

Effects on continuity of care

Given claims made by commercial providers that the 'telephone-first' approach can improve continuity of care for patients, interviewees were asked specifically about changes in the ease with which they were able to see a preferred GP. Again, there was variation between participants in their responses. Some patients reported finding it easier to see or speak to their preferred GP than with the previous system, as a result of the way in which calls were allocated within the practice, with patients being able to specify which GP they would like to call them back. If this was not possible, they could request a face-to-face appointment with the preferred GP during the telephone call. Others, however, reported the opposite, and found it harder to see their GP of choice, observing a trade-off between being seen or spoken to quickly and seeing their preferred GP. These observations highlighted differences between practices introducing the 'telephone-first' approach with respect to their capacity to preserve or enhance continuity of care.

In addition, the degree to which the patients and carers interviewed were concerned about the impact of the introduction of the 'telephone-first' approach in this regard, varied between patients, according to the value they placed on their relationship with a particular GP. Concern was expressed about whether an unfamiliar GP could effectively assess an issue over the telephone and some patients worried about the lack of opportunity to develop or sustain a relationship with a GP (a particular concern among patients with chronic conditions and those with ongoing mental health issues):

> *[an unknown] GP rang me back and I wasn't sure whether he knew anything about me. I'm quite sure he'd looked at my records very briefly but I was*

For peer review only - http://bmjopen.bmj.com/site/about/guidelines.xhtml

> *concerned because it's quite complicated and my preferred GP knows from day one and has worked with me and referred me and supported me, so I didn't know how much this person knew and I just was a little bit unsure and a little bit anxious about whether or not he knew enough about me* (110_1095 – Female in her 60s, part-time work, ongoing mental health issues)

Implications for patient safety

Patients speculated on the implications of the 'telephone-first' approach with respect to patient safety. Views on the impact (or potential impact) of the approach in this regard varied considerably between patients and across practices. While some patients felt vulnerable because of difficulties getting through to the practice by telephone or the fear that diagnoses might be missed in telephone consultations, others thought the approach was safer for patients, in part, because of the considerable reduction in waiting times for appointments:

> *Well I think you get to talk to your doctor when you need to talk to him or her, rather than having a long wait and perhaps getting progressively worse. Certainly if it's an acute condition, it can make a difference, can't it?* (100_1004 - Female patient in her early seventies, retired, multiple chronic health issues)

> *So I phoned up and it was early in the morning and I mentioned to the receptionist what the problem was, and so within minutes another doctor phoned back and he said you, had better come down.* (117_1029 - Female patient in her 60s with chronic health issues, not in employment, caring responsibilities)

Concerns were expressed among patients who were currently confident in their own communication skills that being less articulate or lacking the confidence to push for a face-to-face appointment when required may put some patients at risk of not receiving treatment they needed.

Concerns regarding confidentiality

Concerns regarding confidentiality associated with the 'telephone-first' approach marked a common theme in the analysis, as the system generally required the receptionist to ask the patient for brief details of their problem during the initial call to the practice:

> *you know that whatever you say to a doctor is going to stay with the doctor, with the receptionist, you are never quite sure if it's going to stay there* (117_1066 - Single mother in her 30s, part-time work, infrequent user of GP)

Strong feelings were expressed on this subject, with, for example, one patient describing the approach as '*absolutely disgusting' (103_1042)* .Concern was even expressed by patients who acknowledged the benefit of providing the information in order for calls to be prioritized. Patients also reported concerns about confidentiality associated with the telephone consultation itself, especially if they had to receive the call back from the GP at a time and/or in a location where their

For peer review only - http://bmjopen.bmj.com/site/about/guidelines.xhtml

conversation could be overheard, whether at home with family members present, in a work setting or on public transport.

The importance of understanding the purpose of the approach and how it works

Patients described their understanding of the rationale for the introduction of the ‘telephone-first’ approach, how it was supposed to work in practice and how this had influenced their response to it. The degree to which patients reported that they had been consulted (or at least informed) ahead of the introduction of the new approach, varied considerably. Some patients highlighted their lack of awareness about how the approach would work in practice at the outset, and expressed irritation with a lack of consultation around its introduction, which had resulted in confusion, anxiety and misconceptions regarding the purpose behind the introduction of the approach. Others, however, commented that their initial misgivings had not, by and large, been realized.

Patients indicating their awareness that the approach involved the prioritization of calls according to need, acknowledged the necessity of waiting for a call-back accordingly:

> *I mean sometimes if he’s [the GP] really busy, you don’t hear from him for a couple of hours but then he’s obviously got patients there that are a priority. They know how to prioritize them which is good.* (102_1014 – Female patient, in her late 70s, retired, multiple chronic conditions)

Assessing the overall acceptability of the approach

The advantages and disadvantages of the ‘telephone-first’ approach reported by patients varied between individuals and reflected both the way in which the 'telephone-first’ approach had been implemented and the patients’ own individual characteristics and resources. In assessing the overall acceptability of the approach, patients made reference to both these types of characteristic, and there was apparent interplay between them, with patients explaining how specific issues or disadvantages resulting from how the approach had been implemented were particularly problematic for them as an individual, as a result of personal characteristics or preferences, or the structure of their daily life. For example, having a long or unpredictable wait for a call back from a GP was an issue for patients unable to access a mobile phone or find a quiet, private place to take a call at work, but a lesser concern for those who were retired or were able to work flexibly. Examples of the kinds of factors considered by the patients interviewed as they assessed the overall acceptability of the ‘telephone-first’ approach are presented in tables 2 and 3.

The value attributed to particular advantages and disadvantages varied significantly, even between patients from the same practice or with similar characteristics. A disadvantage that represented a mild annoyance for one patient could render the approach completely unacceptable for another. For example, one patient experiencing mental health issues described the effect of having to wait a long time for a call back from a GP whilst in a distressed state, and how this had influenced her decision to leave the practice:

> *I was really low and so I think I had to wait a few hours [for a call from the GP] and all that time I was in tears and it still took a couple of hours for the doctor. I thought, “Well, now I can’t be bloody bothered.” (103_1042 –*

For peer review only - http://bmjopen.bmj.com/site/about/guidelines.xhtml

Female patient in her 50s, not in employment, mental and chronic physical health problems)

In addition, interviewees also acknowledged that some issues such as difficulty with getting through to the practice initially, or long waits for a response from the practice were (or had the potential to be) of greater concern in some instances than in others, dependent on the perceived urgency of the issue for which they were seeking care.

In describing their assessment of the overall acceptability of the approach, some patients recounted long lists of annoyances (difficulty getting through on the telephone, confidentiality concerns when talking to receptionists, not being able to book in advance, not liking waiting for the call back) but still concluded that they preferred the new approach because they could speak to a doctor within hours and see them the same day if they needed to (an outcome on which they placed particular value).

System / practice characteristic	Factors influencing patients' assessment of the acceptability of the 'telephone-first' approach
**Capacity of the system to meet demand**	Whether telephone calls to the practice are answered promptly
	Whether there are sufficient appointment slots available for both telephone and face-to-face appointments
**Flexibility of the approach**	Whether advanced booking is available
	Degree to which there is flexibility in the timing offered for the GP to call back or ability to book the time of the call-back.
	Whether patients are required to describe their problem to the receptionist
	Whether adjustments have been made for patients who found difficulty with the approach
**Capacity to preserve or enhance continuity of care**	Whether a choice of GP offered for telephone consultation and subsequent face-to-face appointment
**Extent of patient education / knowledge**	Whether patients were consulted prior to introducing the approach
	Whether clear and updated instructions had been provided on how the system works

Table 2. Practice/system characteristics that influenced patients' assessment of the acceptability of the 'telephone-first' approach

Patient characteristic or resource	Factors influencing patients' assessment of the acceptability of the 'telephone-first' approach

For peer review only - http://bmjopen.bmj.com/site/about/guidelines.xhtml

**Communication skills**	The degree to which they feel able to adequately communicate over the telephone
**Confidence**	The degree to which they feel confident to request the outcome they want
**Flexibility of daily schedule**	The degree to which they are able to accommodate time constraints of the approach e.g. being at home during the day/ retired/ working flexibly
**Access to mobile telephone**	Whether they are easily accessible on a mobile telephone
**Value placed on face-to-face contact with GP**	The value they place on face-to-face contact compared to ease and speed of access to care
**Nature of relationship with GP or surgery**	The value they place on a longstanding, trusting relationship with a GP
**Nature of the reason for contacting the surgery**	Perceptions regarding the urgency of the issue for which they are seeking care

Table 3. Individual characteristics and resources that influenced patients' assessment of the acceptability of the 'telephone-first' approach

For peer review only

Discussion

The study showed that, consistent with our published quantitative analysis of the patient and carer survey in our evaluation[2], patients expressed a wide range of views, often strongly held, on the 'telephone-first' approach. Qualitative interviews allowed us to understand these views in greater depth and to explore some of the reasons behind the different views expressed. The new system clearly suited some patients, (e.g. by allowing them to avoid coming into the surgery) but was problematic for others (e.g. when it was difficult for someone working in an open plan office to take a call-back from the GP). Variation was evident within as well as between the different patient groups we recruited from and appeared to be influenced by the interplay of individual and practice level characteristics. Notably, a substantial proportion of negative comments were about the operation of the scheme itself rather than the principles behind it, for example, difficulty getting through on the telephone or being unable to schedule when the GP would call back. Some practices were able to operate the scheme in a way that met their patients' needs better than others and practices appeared to vary significantly in how they had implemented the approach, according to patients' accounts.

The National Health Service in England has prioritized improving access to care for several years and the 'telephone-first' approach is one attempt to address access problems, while at the same time trying to avoid an increase in practice workload. The finding in this study that the approach has been positively received by many of the patients interviewed, is supportive of previous research indicating that there is considerable potential for using telephone consultations in general practice[5,10]. Our findings also chime to a degree with previous findings suggesting that access is not the main driver

For peer review only - http://bmjopen.bmj.com/site/about/guidelines.xhtml

of patients' satisfaction with their GP practices – with interpersonal aspects of care and helpfulness of receptionists being more important,[11,12] (although our findings suggest that the value placed on the different aspects of care may vary considerably between patients, according to their individual needs and preferences).

The study highlights the need for clinicians and policymakers to take the needs of patients with varying care-seeking and interaction approaches into account when making major changes to the organization of general practice care. This change, while designed to improve access to care and reduce the workload burden on practices, clearly did not meet the needs of all patients and provoked outright hostility in some, particularly among those who struggled to access care at all as a result of issues with how the scheme had been implemented. Practices considering making this change should reflect on how they can make the scheme flexible for patients' needs, how they can make it easy for patients to get through on the telephone, and how they can use the approach to enhance both access and continuity of care, and recognize the need for continued development and adaptation of the approach.

A strength of the study is that the interviews included a wide range of patients and carers from a diverse group of practices, purposively sampled to capture a variety of views on the new approach. However, a limitation is the likelihood that practices operating the 'telephone-first' approach successfully were more likely to participate in the patient survey that provided patients who volunteered to be interviewed. We do not know how the views of patients participating in the study may compare with other patients, including those in practices that have not implemented the 'telephone-first' approach.

Questions that could be addressed by future research are how to develop systems that are flexible enough to meet the needs of all their patients. While a rigid 'telephone-first' approach for all consultations does not do this, we observed practices that were modifying this approach (by for example allowing for some advanced booking of appointments) often on an ongoing basis, to meet the needs of patients as closely as they could. Successful approaches are likely to be different in different practices and more work could be done to identify what works best in different circumstances and to share learning.

<u>Conclusions</u>

The 'telephone-first' approach appears to work well for some patients but others find it much less acceptable. Some of the reported problems related to how the approach had been implemented rather than the 'telephone-first' approach in principle and suggests there may be potential for some of the challenges to be overcome. A range of factors were identified that should be considered by practices planning the approach in order to maximise its acceptability and best meet the needs of patients.

Word count: 5787 words + 346 words in tables

<u>Acknowledgements:</u>

We would like to thank the patients and carers interviewed for this study and those who completed the questionnaires which provided the basis for participant recruitment. We are grateful to the GPs

For peer review only - http://bmjopen.bmj.com/site/about/guidelines.xhtml

and staff at practices taking part in the study for their support with its conduct. We would also like to thank the GPs, practice manager and patients on the study steering group who gave guidance on the design and conduct of the study and all those who attended and contributed to study learning events.

Funding: The study was funded by the National Institute for Health Research (HS&DR Project 13/59/40). Part of the funding was used to pay for data to be extracted from practice records by one of the commercial companies providing management support for the 'telephone-first' approach (GP Access). GP Access had no input into the analysis or interpretation of the data. The study was sponsored by Cambridgeshire and Peterborough Clinical Commissioning Group (CCG), who gave initial approval for the project.

This article presents independent research funded by the National Institute for Health Research (NIHR). The views expressed are those of the author(s) and not necessarily those of the NHS, the NIHR or the Department of Health.

Competing interests: All authors have completed the ICMJE uniform disclosure form at www.icmje.org/coi_disclosure.pdf and declare: no financial relationships with any organisations that might have an interest in the submitted work in the previous three years; no other relationships or activities that could appear to have influenced the submitted work.

Author statement:
All authors (SB, JN, JC, JE, EP and MR) contributed to the conception or design of the work and the interpretation of the findings. MR is PI for the study and had oversight, JN was project lead and SB was project manager. SB, JN, JC, JE were involved in data collection. SB, JN, JC, JE and EP conducted data analysis. All authors were involved in drafting and commenting on the paper and have approved the final version.

Ethical approval: The study was approved by the West of Scotland NHS Research Ethics Service (7th May 2015, REC reference 16/WS/0088).

Data sharing:
No additional data are available.

References

[1] Hobbs FD, Bankhead C, Mukhtar T, Stevens S, Perera-Salazar R, Holt T, et al. Clinical workload in UK primary care: a retrospective analysis of 100 million consultations in England, 2007-14. Lancet 2016;387:2323-30. https://doi.org/10.1016/s0140-6736(16)00620-6

[2] NHS England. High quality care now and for future generations: Transforming urgent and emergency care services in England. Page 35-36. https://www.england.nhs.uk/wp-content/uploads/2013/06/urg-emerg-care-ev-bse.pdf

For peer review only - http://bmjopen.bmj.com/site/about/guidelines.xhtml

[3] Newbould J, Abel G, Ball S, Corbett J, Elliott M, Exley J, Martin A, Saunders C, Wilson E, Winpenny E, Yang M, Roland M. Evaluation of telephone first approach to demand management in English general practice: observational study. BMJ 2017; 358: j4197 www.bmj.com/content/bmj/358/bmj.j4197.full.pdf

[4] Hallam L. Access to general practice and general practitioners by telephone: the patient's view. British Journal of General Practice 1993; 43: 331-335. www.ncbi.nlm.nih.gov/pmc/articles/PMC1372558/pdf/brjgenprac00041-0023.pdf

[5] McKinstry B, Watson P, Pinnock H, Heaney D, Sheikh A. Confidentiality and the telephone in family practice: a qualitative study of the views of patients, clinicians and administrative staff. Family Practice 2009; 26: 344-350 https://academic.oup.com/fampra/article/26/5/344/635397

[6] Brant, H, Atherton H, Ziebland S, McKinstry B, Campbell J, Salisbury C. Using alternatives to face-to-face consultations: a survey of prevalence and attitudes in general practice. British Journal of General Practice 2016; 66: e460-466. https://www.ncbi.nlm.nih.gov/pmc/articles/PMC4917048/

[7] Pereira Gray D, Wilkie P. Patient perspectives on the telephone first system. BMJ 2017; 359: j4925. https://doi.org/10.1136/bmj.j4925

[8] Boyatzis R. Transforming qualitative information: Thematic analysis and code development. Thousand Oaks, London, & New Delhi. 1998. SAGE Publications.

[9] O'Brien BC, Harris IB, Beckman TJ, Reed DA, Cook DA. Standards for reporting qualitative research: a synthesis of recommendations. Acad Med. 2014;89(9):1245-1251.

[10] Campbell J, Fletcher E, Britten N, Green C, Holt T, Lattimer V et al. Telephone triage for management of same-day consultation requests in general practice (the ESTEEM trial): a cluster-randomised controlled trial and cost-consequence analysis. Lancet 2014; 384: 1859-68. https://www.thelancet.com/pdfs/journals/lancet/PIIS0140-6736(14)61058-8.pdf

[11] Paddison CA, Abel GA, Roland MO, Elliott M, Lyratzopoulos G, Campbell J. Drivers of overall satisfaction with primary care: evidence from the English General Practice Patient Survey. Health Expectations 2013; 18: 1081–1092 https://onlinelibrary.wiley.com/doi/abs/10.1111/hex.12081

[12] Cowling T, Majeed A, Harris M. Importance of accessibility and opening hours to overall patient experience of general practice: analysis of repeated cross-sectional data from a national patient survey. British Journal of General Practice 2018; 18th June. http://bjgp.org/content/early/2018/06/18/bjgp18X697673?ct=

For peer review only - http://bmjopen.bmj.com/site/about/guidelines.xhtml

Appendix 1. Patient interview guide

This was a broad guide which was developed iteratively as early findings were reviewed by members of the research team

Introduction:

Please can you tell me a little bit about yourself, including whether you work

How long have you been at the practice?

How is your health in general? How regularly do you need access the GP?

Process of booking an appointment:

How do you make an appointment with your GP?

Has this changed since you have been a patient at the practice? (What used to happen?)

Have you had to call the surgery on behalf of someone else / or has someone else called on your behalf? How did that work?

When you call the surgery, how long does it take to get through? Have you experienced any difficulty in getting through?

Do you have to provide any information to the reception staff concerning the call? [could prompt here for how comfortable they feel speaking to receptionist?]

If offered a call back do you get it within a reasonable time period? Do you get to specify when you would like the call back? Have you had any difficulties around the call back?

Is it always a doctor that calls you back? If you have a preferred doctor, do you get to speak to them? [prompt on continuity of care]

Have there been any changes in how the telephone consultations have been run since they were introduced? Has that improved the service/ made it more difficult for you?

Your experience with telephone consultations:

How have you found the experience of using this system?

Is there anything about your lifestyle that makes it more or less difficult for you to use? [prompt: working hours, caring responsibilities etc]

What was the outcome of your last telephone consultation with the GP? (led to a face-to-face appointment, directed to another health-care professional such as nurse, or another service eg. Social services etc. or just given advice over the phone)

If not offered a face-to face appointment with GP:

Were you happy with this outcome? Do you feel the service was satisfactory? Did you seek care elsewhere instead? (e.g. at A&E)

For peer review only

If offered a face-to-face appointment:

If you have a preferred doctor, did you get to see him/her?

Have you noticed any change in the actual appointment [get more time with GP, GP better prepared etc]? Has the quality of the appointment changed?

Have you had other telephone consultations where something different happened? What was the outcome? [repeat prompts as above])

How you feel about the system and what it means for you:

What do you like about the current system for making appointments? ([prompt on convenience, chance to speak to GP when don't want appointment – reassurance, practice seems more organised, get an appointment when want one, appointments not being booked by people for unsuitable use]

What do you dislike about the current system for making appointments? [don't get an appointment when want one, can't communicate well using phone – lost personal connection, elderly – loss of social contact, safety concerns]

Is there anything you miss about the old system (e.g. being able to plan in advance)?

How do you feel about talking to the doctor on the phone? [prompts:Do you feel comfortable talking about your medical condition /do you have any concerns over confidentiality? Do you feel that you are able to make yourself understood?]

How did you feel about the system when it was first introduced? Has this changed over time? [i.e. have they got used to the system]

Has the telephone appointment system changed the way you seek health care services? [prompts: are they thinking about alternative services more before contacting GP? i.e. is this right for the GP should I be going to nurse or don't bother go straight to A&E?]

Has your contact with the GP surgery changed? (i.e. ring more often as know can speak to doctor etc). [prompt on whether offered an appointment, if not with GP who with? Just given advice over phone etc]

Concluding:

If you had the choice would you go back the old system or keep the new system? Why would you make that choice?

Do you think other patients share your view?

Overall are you happy with the care you receive at the practice?

Is there anything else about the telephone appointment system that you'd like to discuss that we haven't spoken about?

Thank you for taking the time to meet with me today

For peer review only

Appendix 2: Characteristics of participants and the 'telephone first' approach

Specific characteristics of the 'telephone first' approach (from patient perspective)	ID	Age[1]	Gender[1]	Ethnicity[1]	Health status[1]	Employment status, nature of health concerns and frequency of GP use[1 2]	Approach preference[2]
**Practice 100 (urban, list size 5,000-9,999)**<br>**Notable features:** no advance booking of face-to face appointments; patient can specify time for call-back; nurse practitioner triages some requests; choice of GP offered for call-back and face-to-face appointment; duty GP takes phone calls in reception office<br>**Problems identified:** more difficult to see GP of choice on the day; can be difficult to get through to reception on Monday mornings<br>**Previous system:** ring up to book in advance or queue up for same day appointments; same-day appointments often not available	100_1004	71	Female	white British	fair	Retired; multiple chronic health issues; frequent user of GP	'Telephone first'
	100_1006	79	Male	white British	good	Retired; multiple chronic health issues, infrequent user of GP	'Telephone first'
	100_1064	Adult	Female	white British	fair	Carer for 85 year old mother with dementia; both have chronic health issues, frequent user of GP;	'Telephone first'
	100_1086	63	Male	white British	good	Recently retired; infrequent user of GP	'Telephone first'
**Practice 101 (urban, list size 5,000-9,999)**<br>**Notable features:** possible to book telephone consultation in advance if preferred GP not available on the day; individual call back lists for each GP; prompt call-back or patient can specify time; some advance booking of face-to face appointments (for follow-ups or if patient unable to make same day appointment); nurse practitioner triages some requests<br>**Problems identified:** can sometimes be difficult to get through to reception<br>**Previous system:** ring up to book in advance; waited 2-3 days for appointment or longer for preferred GP	101_1002	76	Male	white British	very good	Retired; minor health issues requiring specialist input, infrequent user of GP; hearing impairment	'Telephone first'
	101_1006	65	Male	white British	very good	Full time carer for spouse; ongoing health issue requiring specialist input, infrequent user of GP	'Telephone first'
	101_1024	50	Female	other black	fair	Early retirement due to ill health; frequent user of GP	'Telephone first'
	101_1086	37	Male	white British	good	Works full time; ongoing mental and physical health issues; regular review by GP	'Telephone first'
**Practice 102 (urban, list size <5,000)**<br>**Notable features:** quick response from reception to incoming calls; wait for call-back depends on urgency of the issue; some advance booking of follow-up appointments; nurse does some telephone consulting; some forward booking by GPs, patient can always see GP face-to-face if they wish – practice considering making further modifications.<br>**Problems identified:** can sometimes be difficult to get through to reception but this is variable<br>**Previous system:** ring up to book in advance; often waited 3-4 days for appointment but same day appointments available when required	102_1014	77	Female	white British	fair	Retired; multiple chronic conditions; frequent user of GP	'Telephone first'
	102_1019	67	Male	white British	poor	Retired; multiple chronic conditions; regular user of GP; seeing a specialist; lives alone	Conventional
	102_1031	47	Female	white British	poor	Works part time; ongoing mental and physical health issues; frequent user of GP; hearing impairment	'Telephone first'
	102_1064	65	Female	white British	good	Retired; infrequent user of GP	'Telephone first'

For peer review only - http://bmjopen.bmj.com/site/about/guidelines.xhtml

**Practice 103 (urban, list size 5,000-9,999)**<br>**Notable features:** receptionist asks patient whether issue is urgent – call backs prioritised dependent on urgency of issue; flexibility in scheduling call back – patient can request a call back on another day if preferred GP is not in; no advance booking of face-to-face appointments<br>**Problems identified:** can be difficult to get through to reception – phone line sometimes goes dead; face-to-face appointments not available if call later in the day requiring patient to call again the following day<br>**Previous system:** walk-in system for on the day appointments or book by phone – 2/3 days wait	103_1030	41	Female	white British	fair	Mother of two disabled children; frequent user of GP often for advice by phone	'Telephone first'
	103_1034	78	Male	white British	fair	Retired; very frequent user of GP	'Telephone first'
	103_1042	50	Female	white British	no response	Does not work; mental and chronic physical health problems; frequent user of GP	Conventional
	103_1053	71	Female	white British	good	Retired; frequent user of GP	'Telephone first'
	103_1074	67	Female	white British	fair	Retired; infrequent user of GP	'Telephone first', though with modifications
**Practice 104 (urban, list size <5,000)**<br>**Notable features:** receptionist asks patient for a reason for the call -GP reviews list of reasons given and offers face-to-face appointments to some patients on basis of this information alone (without speaking to patient directly); call-back within an hour by GP or by receptionist to call in for a face-to-face appointment<br>**Problems identified**: can be difficult to get through to reception on the phone on a Monday<br>**Previous system:** walk-in system	104_1070	54	Female	white British	fair	Does not work due to chronic health problems; infrequent user of GP as condition well controlled	'Telephone first'
	104_1087	74	Female	white British	good	Retired; increasing frequency of GP visits with age	'Telephone first'
**Practice 105 (urban, list size ≥ 10,000)**<br>**Notable features:** call-back within 30 minutes for urgent issues (wait for call-back depends on urgency); cut off time for patients to call by in order to receive same day call back (e.g. 16.30); nurse triage for some requests; choice of GP offered for call-back and face-to-face appointment; reception spread calls across all GPs, set number of calls per GP per day then a pooled list.<br>**Problems identified:** can be difficult to get through to reception on the phone; online booking no longer available<br>**Previous system:** booking in advance by phone – no difficulty getting an appointment but up to three week wait for non-emergency appointment; on line booking facility	105_1040	79	Female	white British	good	Retired; chronic health issues; frequent user of GP; hearing impairment	'Telephone first'
	105_1043	Adult[3]	Female	white British	n/a	Does not work – mother of young child; chronic health issues (self and child); frequent user of GP	'Telephone first'
	105_1090	78	Male	white British	fair	Retired; multiple chronic health issues; frequent user of GP	'Telephone first'
	105_1099	78	Male	white British	very good	Retired; fit and active; infrequent user of GP	'Telephone first'

**Practice 106 (urban, list size ≥ 10,000)**<br>**Notable features:** variable wait for call-back (from almost instant to many hours); choice of GP offered for call-back and face-to-face appointment; some flexibility for GP to book appointment for next day but no advance booking by reception (e.g. follow-up appointments); patients can choose time for call back.<br>**Problems identified:** can be difficult to get through to reception on the phone on a Monday; can wait all day for a call back<br>**Previous system:** booking in advance by phone – was beginning to get more difficult to get an appointment	106_1013	53	Female	white British	nr	Works flexibly from home; chronic health issue; anxiety; frequent user of GP; previous missed cancer diagnosis	Conventional
	106_1025	78	Female	white British	fair	Retired; chronic health issue; frequent user of GP	'Telephone first'
	106_1026	45	Female	white British	good	Not currently working due to ill health; infrequent user of GP	'Telephone first'
	106_1064	68	Female	white British	fair	Retired; chronic health issues but infrequent user of GP	'Telephone first'
	106_1077	61	Female	white British	fair	Does not work; mental health and multiple chronic physical health problems; frequent user of GP	'Telephone first'
**Practice 108 (urban, list size <5,000)**<br>**Notable features:** variable wait for call-back (from 30 minutes to many hours); duty GP takes calls all day, others only 8-11am; no advance bookings; recorded message indicates cut off time after which only emergency cases will receive a call back (e.g. 15.00)<br>**Problems identified:** variable reports regarding difficulty getting through on the phone; no longer offered choice of preferred GP; can wait all day for a call back; same-day call back not always available<br>**Previous system:** booking in advance by phone – was beginning to get more difficult to get an appointment	108_1032	59	Female	white British	good	Works full time but easy to take calls or make appointments; chronic condition; carer for elderly parents (with hearing impairment); frequent user of GP for self and as carer;	'Telephone first'
	108_1090	66	Female	white British	good	Retired; infrequent user of GP	Conventional
	108_1099	28	Female	Chinese	good	Student – some difficulty taking calls or making appointments; speaks English as a second language; unfamiliar with UK health system; frequent contact with GP	N/a (only experienced this system)
**Practice 110 (urban, list size 5,000 – 9,999)**<br>**Notable features:** phone lines shut off early in the day with recorded message to call the following day; no advance booking available; time of call-back not indicated; separate walk in system also reported to be in operation (bypassing phone system)<br>**Problems identified:** extreme difficulty getting through on the phone; if patient gets through appointments are often unavailable and patient is asked to call the following day; no longer offered choice of preferred GP; can wait all day for a call back;<br>**Previous system:** Advance booking system with long wait of a week or sit and wait on the day. Previously had online system but scrapped.	110_1007	60	Female	white British	fair	Early retirement due to ill health; frequent user of GP	Conventional
	110_1026	74	Male	white British	poor	Retired; multiple chronic conditions requiring specialist input; mental health issues; lives alone; reports limited user of GP due to Telephone	Conventional
	110_1095	63	Female	white/black	fair	Part time/voluntary work; ongoing mental health issues; reports limited user of GP due to Telephone	Conventional

**Practice 112 (urban, list size 5,000 – 9,999)**<br>**Notable features**: receptionist asks for brief details of issue – patient either put straight through to GP or receives very prompt call back; no advance booking available; separate system for nurse appointments<br>**Problems identified:** difficulty getting through on the phone – might take up to an hour; if patient calls after 9 am call backs are often unavailable and patient asked to call the following day; long wait in the surgery for booked appointment<br>**Previous system:** turn up at 8:00am and sit and wait on the day.	112_1015	65	Female	white British	good	Retired; infrequent user of the GP	'Telephone first'
	112_1046	Adult[3]	Male	other	n/a	Working parent,; speaks English as a second language	'Telephone first'
**Practice 114 (urban, list size 5,000 – 9,999)**<br>**Notable features:** receptionist does not ask about the nature of the issue (change from original system); receptionist provides indication of time for call-back and can schedule flexibly around patient's requirements; advance booking available for some follow-up appointments; nurse practitioner does some telephone consulting<br>**Problems identified**: system functioning well<br>**Previous system:** ring to book face-to-face appointment same day appointments were always available if required.	114_1008	48	Male	white British	good	Works/easy to take calls or make appointments; chronic health issues; frequent user of GP	'Telephone first'
	114_1029	Adult[3]	Female	white British	n/a	Carer for elderly father; works from home; frequent user of GP for self and as carer	'Telephone first'
	114_1058	72	Female	white British	poor	Retired; chronic health issues; frequent user of GP	Conventional
**Practice 117 (urban, list size 5,000 – 9,999)**<br>**Notable features:** prompt call-back from GP (often within 10-15 minutes – maximum 1 hour 30 minutes); no advance booking of face-to-face appointments; if preferred GP is not available patient offered choice to speak to a different GP or ring back when available; call back only available for emergencies after 16.00<br>**Problems identified:** time cut off to ensure face-to-face appointment available on the day is unclear;<br>**Previous system:** same day appointment system - rang on the day and had to see whoever was available that day or ring the next day. Sometimes a long wait to see Dr of choice	117_1027	51	Female	white British	very good	Works/difficult to take calls; infrequent user of GP;	'Telephone first'
	117_1029	60	Female	white British	poor	Does not work due to ill health and caring responsibilities; multiple chronic conditions; very frequent user of GP	'Telephone first'
	117_1066	32	Female	white British	good	Single mother/part time voluntary work; infrequent user of GP	'Telephone first'
	117_1073	86	Male	white British	good	Retired; recent hospital stay but previously in good health; infrequent user of GP	'Telephone first'

Standards for Reporting Qualitative Research (SRQR)

Title and abstract
S1 Title Concise description of the nature and topic of the study Identifying the study as qualitative or indicating the approach (e.g., ethnography, grounded theory) or data collection methods (e.g., interview, focus group) is recommended

The nature / topic of the study is included.

S2 Abstract Summary of key elements of the study using the abstract format of the intended publication; typically includes background, purpose, methods, results, and conclusions

These are all included in the abstract.

Introduction
S3 Problem formulation Description and significance of the problem/phenomenon studied; review of relevant theory and empirical work; problem statement

The nature and significance of the problem is described along with a review of empirical work.

S4 Purpose or research question Purpose of the study and specific objectives or questions

The objectives of the study are stated in the last sentence of the introduction.

Methods
S5 Qualitative approach and research paradigm Qualitative approach (e.g., ethnography, grounded theory, case study, phenomenology, narrative research) and guiding theory if appropriate; identifying the research paradigm (e.g., postpositivist, constructivist/ interpretivist) is also recommended; rationale (The rationale should briefly discuss the justification for choosing that theory, approach, method, or technique rather than other options available, the assumptions and limitations implicit in those choices, and how those choices influence study conclusions and transferability. As appropriate, the rationale for several items might be discussed together).

See response S6 below

S6 Researcher characteristics and reflexivity Researchers' characteristics that may influence the research, including personal attributes, qualifications/experience, relationship with participants, assumptions, and/or presuppositions; potential or actual interaction between researchers' characteristics and the research questions, approach, methods, results, and/or transferability

The approach was principally narrative led by a flexible interview guide which is reproduced in appendix 1. However, as we outline in the paper, the topic guide was developed iteratively, both allowing flexibility within the interview for patients to express their own concerns and to introduce elements which had arisen from preliminary analysis of earlier interviews.

S7 Context Setting/site and salient contextual factors; rationale

These are described in the methods and results.

For peer review only

S8 Sampling strategy How and why research participants, documents, or events were selected; criteria for deciding when no further sampling was necessary (e.g., sampling saturation); rationale

The sampling method is described in the method section, including possible biases that may have been introduced by the method of selecting practices.

S9 Ethical issues pertaining to human subjects Documentation of approval by an appropriate ethics review board and participant consent, or explanation for lack thereof; other confidentiality and data security issues

This study was reviewed and given a favourable opinion by the West of Scotland Research Ethics Committee 5 (reference: 15/WS/0088).

S10 Data collection methods Types of data collected; details of data collection procedures including (as appropriate) start and stop dates of data collection and analysis, iterative process, triangulation of sources/methods, and modification of procedures in response to evolving study findings; rationale

These are included in the method section

S11 Data collection instruments and technologies Description of instruments (e.g., interview guides, questionnaires) and devices (e.g., audio recorders) used for data collection; if/how the instrument(s) changed over the course of the study

These are described in the methods section

S12 Units of study Number and relevant characteristics of participants, documents, or events included in the study; level of participation

These are described in the results with further detail provided in a supplementary file

S13 Data processing Methods for processing data prior to and during analysis, including transcription, data entry, data management and security, verification of data integrity, data coding, and anonymization/deidentification of excerpts

These are described in the methods section

S14 Data analysis Process by which inferences, themes, etc., were identified and developed, including the researchers involved in data analysis; usually references a specific paradigm or approach; rationale

This is described in the method section, including the iterative development of the interview guide and the procedure for checking coding.

S15 Techniques to enhance trustworthiness Techniques to enhance trustworthiness and credibility of data analysis (e.g., member checking, audit trail, triangulation); rationale

This is described in the method section (multiple coding, regular discussion of emerging findings in the research team).

For peer review only

Results/findings

S16 Synthesis and interpretation Main findings (e.g., interpretations, inferences, and themes); might include development of a theory or model, or integration with prior research or theory

The results section includes both the main findings and a synthesis of factors affecting the acceptability of the telephone-first approach.

S17 Links to empirical data Evidence (e.g., quotes, field notes, text excerpts, photographs) to substantiate analytic findings

The results section contains quotations from patient and carer interviews to substantiate the findings

Discussion

S18 Integration with prior work, implications, transferability, and contribution(s) to the field. Short summary of main findings; explanation of how findings and conclusions connect to, support, elaborate on, or challenge conclusions of earlier scholarship; discussion of scope of application/ generalizability; identification of unique contribution(s) to scholarship in a discipline or field

These are included in the discussion.

S19 Limitations Trustworthiness and limitations of findings

These are included in the discussion and also in the bullets which follow the abstract.

S20 Conflicts of interest Potential sources of influence or perceived influence on study conduct and conclusions; how these were managed

A conflict of interest statement has been completed by all authors.

S21 Funding Sources of funding and other support; role of funders in data collection, interpretation, and reporting

The source of funding and the role of funders is included.

For peer review only
